# Machine Learning Approaches to Identify Patient Comorbidities and Symptoms That Increased Risk of Mortality in COVID-19

**DOI:** 10.3390/diagnostics11081383

**Published:** 2021-07-31

**Authors:** Sakifa Aktar, Ashis Talukder, Md. Martuza Ahamad, A. H. M. Kamal, Jahidur Rahman Khan, Md. Protikuzzaman, Nasif Hossain, A. K. M. Azad, Julian M. W. Quinn, Mathew A. Summers, Teng Liaw, Valsamma Eapen, Mohammad Ali Moni

**Affiliations:** 1 Department of Computer Science and Engineering, Bangabandhu Sheikh Mujibur Rahman Science and Technology University, Gopalganj 8100, Bangladesh; sakifa.cse@bsmrstu.edu.bd (S.A.); martuza.cse@bsmrstu.edu.bd (M.M.A.); protikkhan28@gmail.com (M.P.); 2 Statistics Discipline, Khulna University, Khulna 9208, Bangladesh; ashistalukder3168@ku.ac.bd; 3 Department of Computer Science and Engineering, Jatiya Kabi Kazi Nazrul Islam University, Trishal, Mymensingh 2220, Bangladesh; kamal@jkkniu.edu.bd; 4 Health Research Institute, University of Canberra, Canberra, ACT 2617, Australia; jahidur.khan@canberra.edu.au; 5 School of Tropical Medicine and Global Health, Nagasaki University, Nagasaki 852-8523, Japan; nasif.stat.iu@gmail.com; 6 Faculty of Science, Engineering & Technology, Swinburne University of Technology Sydney, Sydney, VIC 2150, Australia; akm.azad@uts.edu.au; 7 The Garvan Institute of Medical Research, Healthy Ageing Theme, Darlinghurst, NSW 2010, Australia; j.quinn@garvan.org.au (J.M.W.Q.); m.summers@garvan.org.au (M.A.S.); 8 St Vincent’s Clinical School, Faculty of Medicine, University of New South Wales, Sydney, NSW 2010, Australia; 9 School of Health & Rehabilitation Sciences, The University of Queensland, Brisbane, QLD 4072, Australia; siaw@unsw.edu.au; 10 World Health Organization (WHO) Centre on eHealth, School of Public Health and Community Medicine, Faculty of Medicine, University of New South Wales, Sydney, NSW 2052, Australia; v.eapen@unsw.edu.au; 11 School of Psychiatry, Faculty of Medicine, University of New South Wales, Sydney, NSW 2052, Australia

**Keywords:** comorbidities, COVID-19, machine learning, meta-analysis, SARS-CoV-2

## Abstract

Providing appropriate care for people suffering from COVID-19, the disease caused by the pandemic SARS-CoV-2 virus, is a significant global challenge. Many individuals who become infected may have pre-existing conditions that may interact with COVID-19 to increase symptom severity and mortality risk. COVID-19 patient comorbidities are likely to be informative regarding the individual risk of severe illness and mortality. Determining the degree to which comorbidities are associated with severe symptoms and mortality would thus greatly assist in COVID-19 care planning and provision. To assess this we performed a meta-analysis of published global literature, and machine learning predictive analysis using an aggregated COVID-19 global dataset. Our meta-analysis suggested that chronic obstructive pulmonary disease (COPD), cerebrovascular disease (CEVD), cardiovascular disease (CVD), type 2 diabetes, malignancy, and hypertension as most significantly associated with COVID-19 severity in the current published literature. Machine learning classification using novel aggregated cohort data similarly found COPD, CVD, CKD, type 2 diabetes, malignancy, and hypertension, as well as asthma, as the most significant features for classifying those deceased versus those who survived COVID-19. While age and gender were the most significant predictors of mortality, in terms of symptom–comorbidity combinations, it was observed that Pneumonia–Hypertension, Pneumonia–Diabetes, and Acute Respiratory Distress Syndrome (ARDS)–Hypertension showed the most significant associations with COVID-19 mortality. These results highlight the patient cohorts most likely to be at risk of COVID-19-related severe morbidity and mortality, which have implications for prioritization of hospital resources.

## 1. Introduction

As of the end of May 2021, about 169 million cases of SARS-CoV-2 infection have been confirmed globally, and over 3.5 million deaths causally attributed to it [1]. Asymptomatic human-to-human spread remains a challenging aspect of the containment of this virus, unlike previous coronaviruses SARS and MERS, which showed co-occurrence of symptoms with infectiousness [2,3]. COVID-19 epidemiological data suggests elderly people are most at risk of developing severe symptoms [4,5,6,7] although those symptoms and associated mortality events may occur in all age groups. Some of the prominent symptoms may include dyspnoea, cough, fever, fatigue, myalgia, headache, COPD, and CVD [6,7]. Moreover, as the infection worsens, an acute respiratory distress syndrome may also develop that requires intensive care management [8]. Identifying those most at risk of severe symptoms and death remains a research priority to aid early and appropriate allocation of resources and targeted patient management. As more population data is released, predictive and/or analytical methods can be employed to yield such information for patients based on their clinical characteristics.

Reports are emerging that many of the patients most affected by COVID-19 also present with significant comorbidities. A recent study by Richardson et al. [9] describing 5700 confirmed COVID-19 cases reported that many of these patients were suffering from hypertension (56.6%), obesity (41.7%), or type 2 diabetes (33.8%) at the time of their infection; greater than their respective prevalence in the population, which suggests a link to SARS-CoV-2 effects on metabolic and vascular systems. Jutzeler CR et al. [10] reported that older age, male sex, as well as pre-existing diseases conditions like hypertension and diabetes are critical for the mortality of COVID-19 patients. This indicates that the comorbidities an individual has, may provide crucial prognostic information if SARS-CoV-2 infection co-occurs. There is also recent data emergence, which suggests significant heterogeneity in disease presentation [11]. Hu et al. explains a predictive model for longitudinal clinical data and finds warning of early admission, emergency medicines, and survival predictions. Xu et al. [12] described clinical characteristics (including laboratory and chest radiography data) from 62 Chinese COVID-19 patients that differed from those described by Guan et al. in another Chinese region with some other recent studies [13,14,15]. The reasons for this variation in presentations remain unclear, but differences in prevalence of comorbidities (and other clinical features) in different patient cohorts provide one explanation. The nature and strength of comorbidity association with COVID-19 may also provide important clues to how they may clinically interact and how such interaction may be countered.

To address these issues, we used three approaches to analyze the currently available clinical information. Firstly, we conducted a meta-analysis of available retrospective cohort studies of COVID-19 patient data that focused on comorbidity and selected clinical features. Secondly, we also obtained and aggregated a novel COVID-19 dataset from 4,81,289 patients from across 141 different countries [16,17] and identified significant comorbidity associations. Thirdly, we applied machine learning algorithms to this novel aggregated data to classify the died and alive patients according to comorbidities. These three approaches enabled us to thoroughly assess the comorbidities and clinical features that are most significantly associated with mortality in COVID-19 patients.

## 2. Materials and Methods

### 2.1. Meta-Analysis of Published Data

#### 2.1.1. Search Strategy and Study Selection

The meta-analysis was conducted according to PRISMA (Preferred Reporting Items for Systematic Reviews and Meta-analysis) and MOOSE (Meta-analysis of Observational Studies in Epidemiology) guidelines [18,19,20]. Potential and relevant studies were extracted by conducting a systematic search of databases; from 1 January 2019 to 20 April 2020, in PubMed (Medline), Web of Science, EMBASE, and Cochrane Library databases. This study used the following keywords for database screening: “2019-nCoV”, “2019 novel coronavirus”, “COVID-19”, and “clinical characteristics and symptoms of coronavirus”. Databases using comorbidity combinations for all comorbidities studied were also searched with the following structure: “COVID-19 and diabetes”, “COVID-19 and hypertension”, “COVID-19 and COPD”, and related terms. The list of cited references from selected articles was manually screened to identify missing studies, and all articles selected for the meta-analysis were written in English. For this study, articles that described the clinical characteristics of COVID-19 patients were included, particularly symptoms and comorbidities, along with their prevalence and specific information on the distribution of patients on the basis of severity. Key exclusion criteria were: (a) duplicate publications, (b) case reports, reviews, editorials, letters, or (c) studies that failed to provide sufficient information on clinical patient characteristics, and these are screened manually.

#### 2.1.2. Data Extraction for Statistical Analysis

The literature screening also extracted the data independently from the selected studies. Differences in the chosen literature were reconciled by discussion and rescreening procedure. We extracted the following variables: first author name, year of publication, number of patients, age, sex, number of patients suffering severe diseases (note that patients were not stratified based on the degree of comorbidity severity or symptom severity), number of non-severe patients where these were reported, patient survival, patients needing intensive care unit (ICU) support, and the prevalence of multiple symptoms and comorbidities. The definition of “severe” was clearly described in some articles, however not all. We maintained the case definitions as defined by the original authors. The odds ratios (OR) were calculated to describe the severity of clinical symptoms in severe patients compared to non-severe patients. The degree of variability across studies (heterogeneity) was assessed by I^2^ and Cochran’s Q test [21]. Due to the existence of heterogeneity in studies, random-effects models were utilized to estimate the average effect of variables, along with their precision, which can provide a more accurate estimate of the 95% confidence intervals (CI).

### 2.2. Statistical Analysis and Machine Learning Analysis to Aggregate Novel Clinical Data

#### 2.2.1. Data Collection

We obtained publicly available anonymized clinical data that was derived from both non-hospitalized and hospitalized COVID-19 positive patients; patient diagnoses were based on WHO guidelines [22]. The cases were captured between 14 February 2020 and 31 April 2020. Real-time data were collected from open-source COVID-19 data repositories [16,17]. The data obtained came from a total of 4,81,289 individual patient clinical records from 141 countries.

Summary descriptive statistics for this clinical data are shown in Table S5 and the country-wise patients’ descriptions are shown in Table S6. The clinical attributes collected included clinical symptoms and signs, details of any comorbidities, date of admission in the hospital, date of confirmation of COVID-19 caseness, date of death or hospital release, details of other associated disease outcomes, as well as demographic data; the latter included age, gender, travel history, and location (e.g., city, province, and country) of the patient. The nature of the data was as follows—in both data files, symptoms and comorbidities, and age fields were only continuous, and the rest were categorical. Next, the data set was filtered with some selection criteria, e.g., patients who are deceased and recovered, and released from hospitals. We also excluded patients where data relating to their mortality or recovery from infection was not included. The final filtered dataset included 1143 COVID-19 patients with detailed clinical information, of whom 319 were reported as deceased and 824 as recovered.

#### 2.2.2. Selection of Significant Variables

The focus of this study was to analyze the mortality and survival rates in our filtered 1143 patient datasets and to relate these rates to comorbidity incidence. Thus, we considered respondent age (continuous), sex (male, female), travel history, and the commonly occurring comorbidities, both individually and occurring in multiples. The comorbidities studied included cardiovascular disease (CVD), chronic obstructive pulmonary disease (COPD), cerebrovascular disease (CEVD), chronic kidney disease (CKD), chronic lung disease (CLD), neurodegenerative disease, hypertension, diabetes (type 2), malignancies, infectious diseases, surgical history, asthma, and liver disease. Additionally, we included several clinical symptoms for analysis, including the incidence of fever, cough, pneumonia, acute respiratory distress symptoms (ARDS), dyspnea, fatigue, septic shock, headache, myalgia, diarrhea, and nausea. This was done to predict the disease at an early stage and to identify its relationship with the severity or death. We assessed the influence of these variables on the probability of returning a positive diagnosis of SARS-CoV-2 infection.

#### 2.2.3. Statistical Analysis

Continuous variables were summarized by median along with interquartile range (IQR) and compared by utilizing the Mann–Whitney U test [23]. The frequency of categorical variables was presented as a percentage and compared with a chi-square test [24]. Moreover, Fisher’s exact test [25] was applied to low-frequency cells. A two-sided α (type-I error) less than 0.05 was considered as a measure of statistical significance. All statistical analysis was performed in the R statistical computing environment (version 3.6.1).

#### 2.2.4. Machine Learning Algorithms

In this study, we have used six clinically-applicable supervised machine learning algorithms that were applied to identify the minimum number of symptoms and comorbidities that were predictive of COVID-19 infection [26]. These algorithms included Random Forest, Decision Tree, Gradient Boosting Machine (GBM), XGBoost (XGB), Support Vector Machine (SVM), and Light Gradient Boosting Machine (LGBM). We extracted the required variables from the raw data, and then performed data cleaning and scaling to pre-process the collected data. Imputation techniques were used to address the missing (2.2%) age and gender values, in particular, the missing age was imputed using random values selected from the age IQR, and gender was imputed randomly according to male and female ratios present in the full dataset. Data was randomly split into training (80% individuals) and testing (20% individuals) data sets to perform machine learning prediction and validation. We have set the default parameters to the machine learning models without any hyper parameter tuning before fitting the dataset. To measure accuracy, several measures such as precision, recall or sensitivity, F1-score, area under the receiver operating characteristic curve (AUC-ROC), and log loss values were observed. After achieving high accuracy with the model training, we extracted the features with the highest impact on symptoms and comorbidities classifying a positive COVID-19 infection.

## 3. Results

### 3.1. Meta-Analysis of Published Clinical Reports of COVID-19 Disease

**Figure 1:**
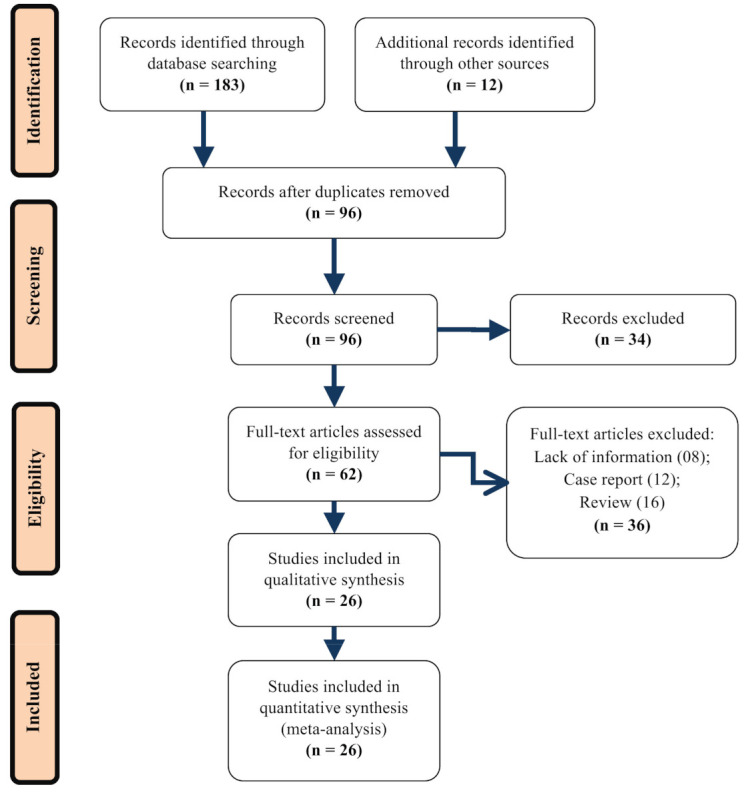
Flow diagram of literature search for including studies in meta-analysis.

Initially, our meta-analysis search terms identified a total of 195 relevant articles. From these articles, we excluded 99 duplicate references and considered the remaining 96. By careful screening of the title and abstract, we excluded 34 articles based on the criteria noted above (e.g., case reports and review reports were ignored) and only considered full-text papers that examined comorbidity and clinical symptoms on COVID-19 patients as listed in Table 1. Finally, for the remaining articles, we reviewed the full text and further removed 36 studies as they were either reviews or clinical details lacking editorials. A total of 26 articles eventually met the inclusion criteria for our meta-analysis. A flow diagram of literature screening is shown in Figure 1.

A total of 13,400 COVID-19 patients from the above-mentioned 26 studies [9,12,13,27,28,29,30,31,32,33,34,35,36,37,38,39,40,41,42,43,44,45,46,47,48] were thus included in our meta-analysis. Most of the studies were conducted in China (24), one was from the USA, and another was from Italy. The mean age of the full sample was 54.5 years, with 8149 (60.81%) males and 39.19% females (Table 1). Of these, there were 2964 patients (22.11%) who developed a severe condition or who were admitted to the ICU or who had died (Table 1). Note that, for calculating the prevalence we considered the full data set from all 26 publications. However, due to lack of information (patients were not stratified based on the degree of severity), we considered only 11 publications in the analysis to assess the effect of symptoms and comorbidities on COVID-19 disease severity or death.

The results of our meta-analysis show the dominant symptomology in COVID-19 disease. Fever (typically defined by a body temperature above 38.5 °C though sometimes not precisely defined) was the most prevalent feature (88.26%, 95% CI 81.31, and 92.84%) (Table 2). The next most common significant symptom was persistent cough (63.68%, 95% CI 57.49, and 69.45%), followed by excessive fatigue (40.48%, 95% CI 34.49, and 48.77%), dyspnea (26.49%, 95% CI 18.50, and 36.39), anorexia (21.92%, 95% CI 13.50, and 33.56), myalgia (21.01%, 95% CI 15.50, and 27.82), headache (9.84%, 95% CI 7.38, and 13.00), diarrhea (7.60%, 95% CI 4.89, and 11.63), and nausea (6.50%, 95% CI 3.10, and 13.10) (as shown in Table 2).

Hypertension (23.41%, 95% CI 17.63, and 30.63) was the most prevalent comorbidity observed among COVID-19 patients, followed by diabetes (11.84%, 95% CI 8.27, and 18.14), CVD (10.00%, 95% CI 7.68, and 12.93), malignancy (4.09%, 95% CI 3.18, and 5.24), cerebrovascular disease (CEVD; 3.23%, 95% CI 2.02, and 5.13), chronic obstructive pulmonary disease (COPD 3.18%, 95% CI: 2.33, and 4.34), chronic kidney disease (CKD; 2.78%, 95% CI 1.74, and 4.41) and chronic liver disease (CLD 2.50%, 95% CI 1.51, and 4.11) (Table 3); prevalence of smoking was 8.83% (95% CI 4.19, and 17.69) (Table 3). Note that prevalence was estimated using a random-effects model, and significant (p<0.05) high heterogeneities were observed for the estimates, with $I^{2}$ ranging from 79 to 99% (see Table 3).

Table S4 shows the meta-analysis results of the association between symptoms as well as comorbidities in severe and non-severe patients from those articles, where severity, ICU support requirement, or death were reported. When clinical symptoms were stratified according to patient severity, higher odds of dyspnea (OR = 2.43, 95% CI 1.52, and 3.89) were observed in the severe symptom group. Thus, COVID-19 patients with dyspnea have more than two-fold increases of risk of developing severe symptoms. The odds of fever (OR = 1.04, 95% CI: 0.85, and 1.28), cough (OR 1.12, 95% CI 0.91, and 1.38), fatigue (OR 1.14, 95% CI 0.96, and 1.36), anorexia (OR 1.56, 95% CI 0.93, and 2.62), myalgia (OR 0.78, 95% CI 0.54, and 1.13), headache (OR 1.04, 95% CI 0.69, and 1.56), diarrhea (OR 1.14, 95% CI 0.81, and 1.61) and nausea (OR 0.93, 95% CI 0.58, and 1.47) were also found to be higher in COVID-19 patients with severe symptoms.

COPD was found to be the comorbidity feature most significantly associated with high disease severity since the odds ratio of COPD (OR 4.76, 95% CI 2.69, and 8.39) was the highest among all other comorbidities and conditions that were considered. The next most significant comorbidity (or condition) relating to disease severity was CEVD (OR 4.54, 95% CI 2.29, and 8.99) followed by CVD (OR 3.46, 95% CI 2.05, and 5.87), CKD (OR 3.22, 95% CI 1.70, and 6.10), type II diabetes (OR 2.08, 95% CI 1.39, and 3.10), malignancy (OR 2.04, 95% CI 1.02, and 4.07), hypertension (OR 1.81, 95% CI 1.49, and 2.20) and smoking (OR 1.74, 95% CI 1.25, and 2.42).

### 3.2. Publication Bias

In parallel to the meta-analysis of data, we also conducted an analysis of publication bias for all symptoms and comorbidities. Table 4 shows the results of possible publication biases, which were assessed using funnel plots and Egger’s testing (for details, see Figure S3). The results of the Egger’s test (p>0.05) suggest that, except for the symptom of anorexia, there were no significant publication biases seen in the variables analyzed.

### 3.3. Clinical Characteristics of Patients in Aggregated Recently Generated COVID-19 Patient Datasets

Following our meta-analysis of the published literature, we also sought to assess recent COVID-19 clinical case data available from open-source online repositories; this allowed us to apply additional novel predictive machine learning methods to COVID-19 data complementing our meta-analysis of the published literature. Data were obtained from two different large data repositories and processed as detailed in the methods section. Following filtering for case data to include only cases with sufficiently detailed clinical information, as well as case mortality information, we obtained a total of 1143 patient cases for analysis. Table 5 displays summary statistics of these 1143 patients stratified by survival/mortality outcomes. The analysis found that out of the 1143 patients, 86.61% had no comorbidities, whereas 5.34% and 7.87% of patients had only one or more than one comorbidity, respectively. The most common coexisting comorbidities were hypertension (8.66%), diabetes (7.44%), cardiovascular disease (3.5%), and kidney disease (1.75%). In contrast, malignancy of any kind (0.87%), asthma (0.87%), COPD (0.61%), chronic lung disease (0.61%), cerebrovascular disease (0.44%), surgical history (0.26%), neurodegenerative disease (0.17%), infectious disease (0.17%), and liver disease (0.17%) were found to be far less likely to co-occur with COVID-19 in this dataset. Analyzing this data for clinical symptomatology found that the most common clinical presentation of patients with COVID-19 was fever (14.17%) followed by cough (12.42%), pneumonia (6.47%), acute respiratory distress symptoms (5.69%), dyspnea (3.06%), fatigue (2.19%), septic shock (1.49%), headache (0.96%), myalgia (0.79%), diarrhea (0.61%), and nausea (0.26%).

Table 5 also shows the status of patients who were deceased. The selected 1143 patients included 319 (27.91%) as deceased, of which 32.60% were female and 61.76% were male. The median age of the deceased patients was 51 years and IQR of 36 to 66 years. A majority of patients (67.08%) had no comorbidities in this dataset. Only 10.97% of patients had one comorbidity, while 21.94% had more than one comorbidity. In the deceased patient subgroup, the rate of comorbidities was significantly higher than surviving patients. The comorbidities most frequently seen in COVID-19 patients that did not survive their infection included type 2 diabetes (19.12%), cardiovascular disease (6.27%), and kidney disease (4.08%). However, while the other comorbidities we studied (see Table 5) were less frequently observed in COVID-19 patients, when they did co-occur, they did so only in patients who had died (Table 5). Descriptive analysis of the symptoms in the deceased COVID-19 patients found that the most significant symptoms seen in the deceased patients were pneumonia (21.32%), fever (12.85%), cough (11.60%), acute respiratory distress symptom (9.72%), and septic shock (4.70%) (Table 5).

### 3.4. Supervised Machine Learning Identifies the Most Significant COVID-19 Comorbidities

**Figure 2:**
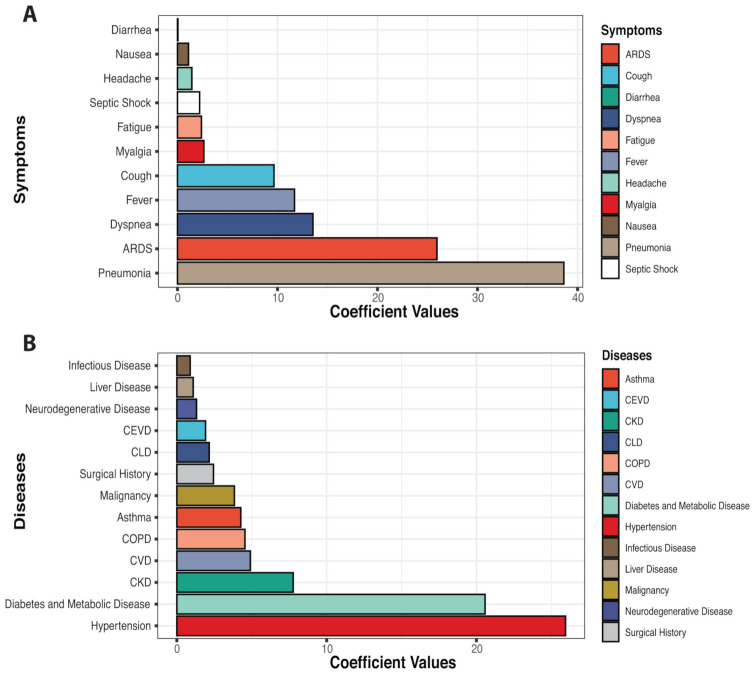
Machine learning models predict the important symptoms and comorbidities that are associated with the severity or death of COVID-19 patients. The high coefficient values of ML model outcomes mean the higher significant association of death. (**A**) represents the significance of symptoms that are linked with death; (**B**) represent the significance of disease comorbidities that are linked with death.

To predict significant COVID-19 comorbidities, and to compare with our meta-analysis of the published literature, we designed and performed a machine learning analysis of our 1143 patients’ datasets. We applied six different machine learning algorithmic approaches (Random Forest, Decision Tree, GBM, XGB, SVM, and LGBM) to identify the best predictors of COVID-19 patient mortality among the comorbidities and symptoms. We achieved a regression accuracy of >80% in all six approaches to comorbidity and mortality; specifically, that was 83% for Decision Tree, 84% for GBM, and 86% for XGB, 87% for Random Forest and SVM, and 88% for LGBM. These methods also achieved accuracy for symptoms of >85% in all six approaches, with GBM and LGBM showing 90% accuracy. Accuracy matrices, including precision, recall or sensitivity, F1-score, area under the curve (AUC-ROC), and log loss values, are shown in Table S1 for symptoms data and in Table S2 for comorbidity data. The coefficient values for the features (symptoms) are reported in Table S3, and the features (comorbidities) are reported in Table S4. Our results indicate that “age” is the most significant predictor of mortality as well as gender. We compared both results (most significant features) for symptoms and comorbidities found from different algorithms and got similar predictions. In Figure 2, we represent the significance level for symptoms and diseases. After calculating the coefficient values for every algorithm, we measured the symptoms and diseases on the same scale by quantile normalization and using the average normalized values in Figure 2. The most significant symptoms were pneumonia, acute respiratory distress syndrome (ARDS), dyspnea, fever, and cough (Table S3) and the most significant comorbidities found were hypertension, diabetes and metabolic diseases, chronic kidney disease, cardiovascular disease, chronic obstructive pulmonary disease (COPD), asthma, and malignancy in this cohort (Table S4).

### 3.5. Significant Pairs of Interacting Comorbidities and Symptoms Associated with Death in COVID-19

**Figure 3:**
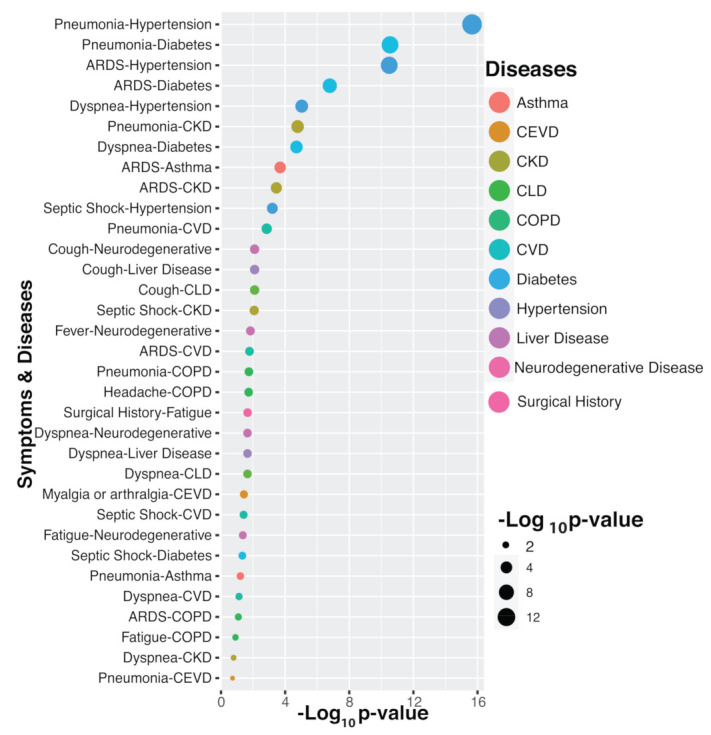
Association and impact of combined symptoms and ccomorbidity interactions in COVID-19 deceased patients.

One of the unique findings of this study is the identification of significant pairs of comorbidities and symptoms that are associated with death among COVID-19 patients. For identification of symptom-comorbidity interactions, we applied the Fisher’s exact testing procedure. The negative logarithm of the *p*-values obtained from the tests is presented in Figure 3. We observed that the symptom–comorbidity combination of Pneumonia–Hypertension, Pneumonia–Diabetes and ARDS–Hypertension had the most significant effects on mortality in COVID-19 patients (Figure 3).

## 4. Discussion

The recent and continuing spread of SARS-CoV-2 has vastly outpaced the ability of many public health care systems around the world to respond and manage. There are many examples from even advanced economies, where medical professionals have had to make distressing decisions about prioritization of insufficient care resources [10,49]. This highlights the critical need for fast and accurate classification of those patients most at risk of severe disease or fatality to best allocate hospital resources during times of crisis [15,50].

To this end, we have performed a number of analyses to assess how disease outcome is related to a range of patient comorbidities and clinical features. Firstly, we investigated published COVID-19 clinical data using a conventional meta-analysis. We found almost no evidence of publication bias in this data, and little grey literature sources of use to our study. This may reflect the current strong imperative to rapidly publish any available studies. Our meta-analysis identified COPD, CEVD, CVD, diabetes, malignancy, and hypertension as most significantly associated with COVID-19 severity in the current published literature.

We also obtained and analyzed aggregated COVID-19 patient data (not derived from published clinical trials or retrospective studies) using statistical and machine learning methods. We found that patients most at risk of dying from COVID-19 had particular comorbidities and patient features, most of which were seen in our meta-analysis. Our machine learning analysis of this patient dataset for the classification of deceased versus recovered COVID-19 patients identified COPD, CVD, CKD, diabetes, malignancy, hypertension, and asthma as most significant. These results provide detailed insights into the strength of the relationship between these factors and patients’ risk of dying from COVID-19, identifying prognostic factors by largely independent means. This may lead to identification of disease mechanisms of interest by considering pathways that may be common to these comorbidities. Already such considerations have been made with several studies reporting strong evidence for a link between SARS-CoV-2 actions and vascular damage [29]. Further, given that the angiotensin converting enzyme (ACE-2) receptor is used by the virus for entry into host cells, it has been suggested that the already strained ACE-2-Ang-(1-7)-Mas in metabolic disorders may result in a respiratory compromise [30]. The role of upregulation of the ACE-2 receptors by ACE inhibitors and angiotensin II receptor blockers used in the management of hypertension, diabetes, and CKD [31] also requires further exploration in elucidating the metabolic pathways that underpin the relationship between these co-morbidities and increased SARS-CoV-2 related severe morbidity and mortality.

It is likely that there are many different factors interacting that lead to the co-incidence of COVID-19 and comorbidities greatly detrimental to patient outcome [2,9,12,27,30]. We found using machine learning classification methods that age and gender are the most significant predictors of COVID-19 mortality. Indeed, it is likely that in many cohorts, age is strongly associated with the co-occurrence of significant comorbidities as these tend to be age-related diseases [51]. Nevertheless, comorbidities analyzed here such as diabetes, hypertension [52] and asthma do occur across age categories, suggesting mortality in COVID-19 is impacted by other characteristics yet to be identified; perhaps differences in environment and/or genetic predispositions are likely relevant factors for future consideration. Moreover, our applied framework could be helpful for the prediction or classification problem utilizing the similar type of data [53,54]. However, such a model must be trained using related data. In contrast, this model would not be applicable for a study employing quite different kinds of datasets. Thus, it could be applicable to identify features or risk factors for any disease comorbidities utilizing available data.

Mechanistically, the association between lung-related comorbidities such as COPD and COVID-19 disease severity is an expected outcome of this study. COPD is a chronic lung condition, often caused by a patient’s history of smoking [55]. Patients with COPD present with pulmonary damage and chronic breathing difficulty; thus, the co-occurrence of a severe lower respiratory viral infection and pneumonia is a significant challenge, particularly in the elderly. In contrast, the association of severe COVID-19 disease with conditions such as vascular diseases (CVD, CEVD) and diabetes, is perhaps more complex. Data are emerging, however, that suggests SARS-CoV-2 infection is associated with a severe inflammatory storm that can result in vascular inflammation, as well as myocarditis. Thus cardio-vascular and metabolic diseases are likely compounding the impact of COVID-19; perhaps presenting a therapeutic opportunity for broad-spectrum anti-inflammatory medications, although the data on efficacy remain to be acquired.

An important consideration remains the limitations of the available data for predictive analyses in the time of the present study. COVID-19 remains a relatively recent phenomenon [50,56,57,58,59,60], and, thus, the data may contain biases that cannot as yet be circumvented. For example, the majority of data coming from mainland China presents biases related to population genetics as well as environmental effects that will not be observed in similar European datasets. Nevertheless, our analysis of this cohort data from 1143 patients comes from repository data acquired from across 141 countries; thus, systematic biases of this kind should be minimal. In machine learning analysis, the cross-validation analysis was not conducted, which can be done in future studies. Additionally, however, there may be unidentified reporting biases in global hospital data due to severe under-resourcing and staff shortages in some locations, necessitating priority reporting. Over the coming months, more data will become available from more diverse nations and population groups that will enable fuller investigation of these issues.

## 5. Conclusions

In summary, we have performed a comprehensive meta-analysis of available published literature, as well as a novel machine learning analysis of a separate cohort of COVID-19 patients. We identified significant comorbidities and COVID-19 patient symptoms that are important for consideration when assessing patient needs; something that remains critical at a time where hospitals are often understaffed and under-resourced. Data suggest that the comorbidities most implicated in severe COVID-19 are lung-related, such as COPD and asthma, as well as vascular-related conditions, such as CVD and CEVD. Thus, it is critical that at-risk populations be prioritized in efforts around social isolation and resource allocation during this pandemic. As data continue to be accrued, it will become possible to answer questions regarding gender and age-related comorbidity relationships including medication history as well as population genetics and environmental effects that may be relevant to treatment optimization.

## Figures and Tables

**Table 1:** Summary of study characteristics reported in the selected publications.

First Author	Study Type	Year of Publication	Country	Sample Size (*n*)	Gender		Mean/Median Age (Years)	Severe or Death Patients *n* (%)	Reference
					Male *n* (%)	Female *n* (%)			
Wang et al.	Retrospective case series	2020	China	138	75 (54.35)	63 (45.65)	56	36 (26.09)	[27]
Richardson et al.	Case series	2020	USA (New York)	5700	3437 (60.30)	2263 (39.70)	63	373 (6.54)	[9]
Xu et al.	Retrospective case series	2020	China	62	35 (56.45)	27 (43.55)	41	NR	[12]
Guan et al.	Case report	2020	China	1099	640 (58.23)	459 (41.77)	47	173 (15.74)	[13]
Guan WJ et al.	Retrospective case series	2020	China	1590	904 (56.86)	686 (43.14)	48.9	254 (15.97)	[28]
Huang et al.	Prospective cohort	2020	China	41	30 (73.17)	11 (26.83)	49	13 (31.71)	[2]
Guo et al.	Retrospective case series	2020	China	187	91 (48.66)	96 (51.34)	58.50	NR	[29]
Zhou et al.	Retrospective cohort	2020	China	191	119 (62.30)	72 (37.70)	56.0	66 (34.55)	[30]
Zhang et al.	Cross-sectional	2020	China	140	71 (50.71)	69 (49.29)	57	58 (41.43)	[31]
Wu et al.	Retrospective case series	2020	China	80	39 (48.75)	41 (51.25)	46.10	NR	[32]
Liu et al.	Retrospective case series	2020	China	137	61 (44.53)	76 (54.47)	57	NR	[33]
Liu J et al.	Prospective cohort	2020	China	61	31 (50.82)	30 (49.18)	40	17 (27.87)	[34]
Chen et al.	Retrospective single-center	2020	China	99	67 (67.68)	32 (32.32)	55.5	NR	[35]
Yang et al.	Retrospective single-center	2020	China	52	35 (67.31)	17 (32.69)	59.7	52 (100.00)	[36]
Wu C et al.	Retrospective cohort	2020	China	201	128 (63.68)	73 (36.32)	51	53 (26.37)	[37]
Jie Li et al.	Cross-sectional	2020	China	17	9 (52.94)	8 (47.06)	45.1	NR	[38]
Liu W et al.	Retrospective case series	2020	China	78	39 (50.00)	39 (50.00)	38	NR	[39]
Mo et al.	Retrospective single-center	2020	China	155	86 (55.48)	69 (44.52)	54	55 (35.48)	[40]
Du et al.	Retrospective case series	2020	China	85	62 (72.94)	23 (27.06)	65.8	NR	[41]
Rong-Hui et al.	Prospective cohort	2020	China	179	97 (54.19)	82 (45.81)	57.6	NR	[42]
Feng et al.	Retrospective case series	2020	China	476	271 (56.93)	205 (43.07)	53	26 (5.46)	[43]
Chen et al.	Retrospective case series	2020	China	274	171 (62.41)	103 (37.59)	62	113 (41.24)	[44]
Grasselli et al.	Retrospective case series	2020	Italy	1,591	1304 (81.96)	287 (18.04)	63	1591 (100.00)	[45]
Deng et al.	Retrospective case series	2020	China	225	73 (32.44)	152 (67.56)	69	NR	[46]
Wang et al.	Retrospective single-center	2020	China	339	166 (48.97)	173 (51.03)	69	65 (19.17)	[47]
Chen TL et al.	Retrospective single-center	2020	China	203	108 (53.20)	95 (76.80)	54	19 (9.36)	[48]
Total		-	-	13,400	8149 (60.81)	5206 (39.19)	-	2964 (22.11%)	-

NR = Not Reported.

**Table 2:** Prevalence of symptoms in COVID-19 patients in the selected studies.

First Author	Year of Publication	Sample Size (*n*)	Clinical Symptoms									Reference
			Fever (%)	Cough (%)	Fatigue (%)	Anorexia (%)	Myalgia (%)	Dyspnea (%)	Diarrhea (%)	Nausea (%)	Headache (%)	
Wang et al.	2020	138	98.55	59.42	69.57	39.86	34.78	31.16	26.09	10.14	6.52	[27]
Richardson et al.	2020	5700	NR	NR	NR	NR	NR	NR	6.54	NR	NR	[9]
Xu et al.	2020	62	77.42	80.65	51.61	NR	51.61	NR	NR	NR	33.87	[12]
Guan et al.	2020	1099	43.04	67.79	38.13	NR	NR	NR	15.74	5.00	13.65	[13]
Guan WJ et al.	2020	1590	84.97	66.16	36.73	NR	NR	NR	15.97	5.03	12.89	[28]
Huang et al.	2020	41	97.56	75.61	43.90	NR	43.90	53.66	31.71	NR	7.32	[2]
Guo et al.	2020	187	NR	NR	NR	NR	NR	NR	NR	NR	NR	[29]
Zhou et al.	2020	191	94.24	79.06	23.04	NR	15.18	NR	34.55	3.66	NR	[30]
Zhang et al.	2020	140	78.57	64.29	64.29	12.14	NR	NR	41.43	17.14	NR	[31]
Wu et al.	2020	80	NR	NR	NR	NR	NR	NR	NR	NR	NR	[32]
Liu et al.	2020	137	81.75	48.18	32.12	NR	32.12	18.98	NR	62.04	9.49	[33]
Liu J et al.	2020	61	98.36	63.93	57.38	NR	NR	4.92	27.87	8.20	34.43	[34]
Chen et al.	2020	99	82.83	81.82	NR	NR	NR	NR	NR	1.01	8.08	[35]
Yang et al.	2020	52	98.08	28.85	NR	NR	3.85	23.08	100.00	NR	1.92	[36]
Wu C et al.	2020	201	93.53	81.09	32.34	NR	32.34	39.80	26.37	NR	NR	[37]
Jie Li et al.	2020	17	70.59	76.47	47.06	NR	23.53	NR	NR	NR	NR	[38]
Liu W et al.	2020	78	NR	43.59	NR	NR	NR	NR	NR	NR	NR	[39]
Mo et al.	2020	155	81.29	62.58	38.71	16.77	NR	1.29	35.48	1.94	5.16	[40]
Du et al.	2020	85	91.76	NR	58.82	56.47	16.47	70.59	NR	NR	4.71	[41]
Rong-Hui et al.	2020	179	98.88	81.56	39.66	NR	18.99	49.72	NR	NR	9.50	[42]
Feng et al.	2020	476	81.93	NR	56.51	NR	11.55	NR	5.46	NR	NR	[43]
Chen et al.	2020	274	90.88	67.52	50.00	24.09	21.90	NR	41.24	8.76	11.31	[44]
Grasselli et al.	2020	1591	NR	NR	NR	NR	NR	NR	100.00	NR	NR	[45]
Deng et al.	2020	225	42.22	20.89	13.33	NR	13.33	34.22	NR	NR	NR	[46]
Wang et al.	2020	339	91.74	52.80	39.82	27.73	4.72	40.71	19.17	3.83	3.54	[47]
Chen TL et al.	2020	203	89.16	60.10	7.88	2.96	26.60	1.48	9.36	1.48	4.93	[48]
Overall prevalence (95% CI)			88.26 (81.31, 92.84)	63.68 (57.49, 69.45)	40.48 (34.49, 48.77)	21.92 (13.50, 33.56)	21.01 (15.50, 27.82)	26.49 (18.50, 36.39)	7.60 (4.89, 11.63)	6.50 (3.10, 13.10)	9.84 (7.38, 13.00)	-
$I^{2}\left(\%\right)$			98	94	94	94	92	93	93	97	87	-
p for heterogeneity			<0.01	<0.01	<0.01	<0.01	<0.01	<0.01	<0.01	<0.01	<0.01	-

Meta-analysis for the prevalence was calculated from random-effects model analysis (see Figure S1 for details); NR = Not Reported.

**Table 3:** Prevalence of comorbidities in COVID-19 patients in the selected studies.

First Author	Year of Publication	Sample Size (*n*)	Comorbidities									Reference
			Hypertension (%)	Diabetes (%)	CVD (%)	Malignancy (%)	COPD (%)	CEVD (%)	CKD (%)	CLD (%)	Smoking (%)	
Wang et al.	2020	138	31.16	10.14	14.49	7.25	2.90	5.07	2.90	2.90	NR	[27]
Richardson et al.	2020	5700	53.09	31.72	14.46	5.61	5.04	NR	7.95	0.19	47.21	[9]
Xu et al.	2020	62	8.06	1.61	NR	NR	1.61	1.61	1.61	11.29	NR	[12]
Guan et al.	2020	1099	15.01	7.37	2.46	0.91	1.09	1.36	0.73	2.09	14.37	[13]
Guan WJ et al.	2020	1590	16.92	8.18	3.71	8.18	1.51	1.89	16.92	1.51	6.98	[28]
Huang et al.	2020	41	14.63	19.51	4.88	2.44	2.44	NR	NR	2.44	7.31	[2]
Guo et al.	2020	187	32.62	14.97	11.23	NR	2.14	NR	3.21	NR	9.62	[29]
Zhou et al.	2020	191	30.37	18.85	7.85	NR	NR	NR	1.05	NR	5.75	[30]
Zhang et al.	2020	140	30.00	12.14	7.14	NR	1.43	NR	1.43	5.71	6.42	[31]
Wu et al.	2020	80	NR	NR	31.25	5.00	NR	NR	1.25	1.25	NR	[32]
Liu et al.	2020	137	9.49	10.22	7.30	1.46	1.46	NR	NR	NR	NR	[33]
Liu J et al.	2020	61	19.67	8.20	NR	NR	8.20	1.64	NR	NR	6.55	[34]
Chen et al.	2020	99	NR	12.12	40.40	NR	1.01	NR	NR	NR	NR	[35]
Yang et al.	2020	52	NR	3.85	7.69	1.92	NR	NR	NR	NR	3.84	[36]
Wu C et al.	2020	201	19.40	10.95	3.98	NR	2.49	NR	1.00	3.48	NR	[37]
Jie Li et al.	2020	17	5.88	NR	NR	NR	NR	NR	NR	NR	17.64	[38]
Liu W et al.	2020	78	10.26	6.41	NR	5.13	2.56	NR	NR	NR	6.41	[39]
Mo etal.	2020	155	23.87	9.68	9.68	4.52	3.23	4.52	3.87	4.52	3.87	[40]
Du et al.	2020	85	37.65	22.35	11.76	7.06	2.35	8.24	3.53	5.88	NR	[41]
Rong-Hui et al.	2020	179	32.40	18.44	16.20	2.23	NR	NR	2.23	NR	NR	[42]
Feng et al.	2020	476	NR	NR	NR	NR	NR	NR	NR	NR	9.24	[43]
Chen et al.	2020	274	33.94	17.15	8.76	2.55	6.57	1.46	1.46	4.01	6.93	[44]
Grasselli et al.	2020	1591	31.99	11.31	14.02	5.09	2.64	NR	2.26	1.76	NR	[45]
Deng et al.	2020	225	17.78	7.56	5.78	2.67	9.78	NR	NR	NR	NR	[46]
Wang et al.	2020	339	40.71	15.93	15.63	4.42	6.19	6.19	3.83	0.59	NR	[47]
Chen TL et al.	2020	203	21.18	7.88	7.88	3.45	3.94	4.43	3.94	3.94	NR	[48]
Overall prevalence (95% CI)			23.41 (17.63, 30.63)	11.84 (8.27, 18.14)	10.00 (7.68, 12.93)	4.09 (3.18, 5.24)	3.18 (2.33, 4.34)	3.23 (2.02, 5.13)	2.78 (1.74, 4.41)	2.50 (1.51, 4.11)	8.83 (4.19, 17.69)	-
$I^{2}\left(\%\right)$			98	97	94	79	82	79	95	88	99	-
p for heterogeneity			<0.01	<0.01	<0.01	<0.01	<0.01	<0.01	<0.01	<0.01	<0.01	-

CVD = Cardiovascular disease; COPD = Chronic obstructive pulmonary disease; CEVD = Cerebrovascular disease; CKD = Chronic Kidney Disease; and CLD = Chronic lung disease. Note: Meta-analysis for the prevalence was calculated from random-effects model analysis (see Figure S1 for details).

**Table 4:** Odds ratio representing the severity of comorbidities and symptoms in COVID-19 patients obtained from meta-analysis of published data.

Outcomes	Number of Studies	Number of Patients	Odds Ratio (95% CI)	$I^{2}\;\%$ (*p* Value)	*p* Value of Egger’s Test
**Comorbidities**	-	-	-	-	-
Hypertension	10	2641	1.81 (1.49, 2.20)	0 (0.72)	0.551
Diabetes	11	2693	2.08 (1.39, 3.10)	46 (0.05)	0.949
CVD	6	1150	3.46 (2.05, 5.87)	32 (0.21)	1.141
Malignancy	6	1161	2.04 (1.02, 4.07)	0 (0.67)	0.466
COPD	8	2176	4.76 (2.69, 8.39)	0 (0.97)	0.235
CEVD	6	2208	4.54 (2.29, 8.99)	16 (0.31)	0.633
CKD	8	2539	3.22 (1.70, 6.10)	0 (0.93)	0.593
Smoking	6	1920	1.74 (1.25, 2.42)	0 (0.88)	0.916
**Clinical Symptoms**	-		-	-	-
Fever	11	2693	1.04 (0.85, 1.28)	42 (0.07)	0.479
Cough	11	2693	1.12 (0.91, 1.38)	41 (0.09)	0.354
Fatigue	10	2641	1.14 (0.96, 1.36)	0 (0.99)	0.183
Anorexia	5	1046	1.56 (0.93, 2.62)	62 (0.03)	0.018
Myalgia	7	1238	0.78 (0.54, 1.13)	0 (0.68)	0.685
Dyspnea	7	989	2.43 (1.52, 3.89)	19 (0.29)	0.774
Diarrhea	9	2600	1.14 (0.81, 1.61)	8 (0.37)	0.731
Nausea	7	2242	0.93 (0.58, 1.47)	15 (0.31)	0.458
Headache	6	1779	1.04 (0.69, 1.56)	11 (0.34)	0.832

Note: CVD = Cardiovascular disease; COPD = Chronic obstructive pulmonary disease; CEVD = Cerebrovascular disease; CKD = Chronic Kidney Disease; CLD = Chronic lung disease; Odds ratio: Meta-Analysis for overall odds ratio (see, Figures S2 and S3 for details); *p* value of Egger’s test: Assessing the publication bias (see, Figure S3 details).

**Table 5:** Association between patient survival and selected demographic characteristics, comorbidities and clinical symptoms.

Characteristics	All Patients, *n* = 1143 (%)	Patient’s Condition		*p* Value
		Dead, *n* = 319 (%)	Survived, *n* = 824 (%)	
**Age, median (IQR)**	51 (36–66)	74 (63–82)	46 (32–53)	<0.001
Gender				<0.001
Female	388 (33.95)	104 (32.60)	284 (34.47)	-
Male	600 (52.49)	197 (61.76)	403 (48.91)	-
Unknown	155 (13.56)	18 (5.64)	137 (16.63)	-
Travel History	370 (32.37)	80 (25.08)	290 (35.19)	0.001
**Comorbidities**				
CVD	21 (1.84)	16 (5.01)	5 (0.61)	<0.001
CEVD	4 (0.35)	4 (1.25)	0	0.005
CLD	7 (0.61)	3 (0.94)	4 (0.49)	0.406
Malignancy	9 (0.79)	4 (1.25)	5 (0.61)	0.275
Diabetes and Metabolic Disease	80 (6.99)	61 (19.12)	19 (2.31)	<0.001
Liver Disease	2 (0.17)	2 (0.63)	0	0.078
CKD	20 (1.75)	13 (4.08)	7 (0.85)	<0.001
Neurodegenerative Disease	2 (0.17)	2 (0.63)	0	0.078
Infectious Disease	2 (0.17)	0	2 (0.24)	1.00
Surgical History	3 (0.26)	1 (0.31)	2 (0.24)	1.00
COPD	8 (0.69)	6 (1.88)	2 (0.24)	0.007
Asthma	10 (0.87)	5 (1.57)	5 (0.61)	0.226
Hypertension	100 (8.74)	74 (23.19)	26 (3.15)	<0.001
**Symptoms**				
Headache	11 (0.96)	1 (0.31)	10 (1.21)	0.308
Fever	145 (12.68)	39 (12.22)	106 (12.86)	0.848
Cough	113 (9.88)	29 (9.09)	84 (10.19)	0.653
Fatigue	25 (2.19)	8 (2.51)	17 (2.06)	0.814
Nausea	3 (0.26)	1 (0.31)	2 (0.24)	1.00
Diarrhea	7 (0.61)	1 (0.31)	6 (0.73)	0.681
Myalgia	11 (0.96)	3 (0.94)	8 (0.97)	1.00
Dyspnea	59 (5.16)	48 (15.04)	11 (1.33)	<0.001
Pneumonia	74 (6.47)	66 (20.69)	6 (0.73)	<0.001
ARDS	67 (5.86)	60 (18.81)	7 (0.85)	<0.001
Septic Shock	18 (1.57)	16 (5.02)	2 (0.24)	<0.001
**Comorbidity Number**				<0.001
No Comorbidity	990 (86.61)	214 (67.08)	775 (94.05)	-
Comorbidity = 1	61 (5.34)	35 (10.97)	27 (3.28)	-
Comorbidity > 1	90 (7.87)	70 (21.94)	5 (0.61)	-

Note: CVD = Cardiovascular disease; COPD = Chronic obstructive pulmonary disease; CEVD = Cerebrovascular disease; CKD = Chronic Kidney Disease; CLD = Chronic lung disease; ARDS = Acute Respiratory Distress Syndrome.

## Data Availability

All the programming codes are available on a GitHub repository and data are available in another GitHub repository [17] and a spreadsheet [16]: https://github.com/m-moni/COVID-19 (accessed on 31 April 2020); https://github.com/beoutbreakprepared/nCoV2019 (accessed on 31 April 2020); and https://docs.google.com/spreadsheets/d/1Gb5cyg0fjUtsqh3hl_L-C5A23zIOXmWH5veBklfSHzg/edit#gid = 447265963 (accessed on 31 April 2020).

## Supplementary Materials

The following are available online at https://www.mdpi.com/article/10.3390/diagnostics11081383/s1, Figure S1. Meta-analysis of prevalence of comorbidities and symptoms COVID-19 fatalities. Figure S2. Meta-analysis of severity of comorbidities and symptoms in COVID-19 fatalities. Figure S3. Assessment of publication bias using funnel plot and Egger’s test. Table S1. Accuracy and Evaluation matrices for symptoms data in ML analysis. Table S2. Accuracy and Evaluation matrices for comorbidity data in ML analysis. Table S3. Coefficient values for each symptom applying after ML methods. Table S4. Coefficient values for each comorbidity applying after ML methods. Table S5. Assessing association between comorbidity and symptoms using Fisher’s exact test of deceased patients. Table S6. The distribution of patients’ according to countries. The following are available on another file.
