# Supplementary material for: Machine Learning Approaches to Identify Patient Comorbidities and Symptoms That Increased Risk of Mortality in COVID-19
Source: Diagnostics (Basel). 2021 Jul 31;11(8):1383. doi: 10.3390/diagnostics11081383 (PMC8393412; doi:10.3390/diagnostics11081383)
Supplement: Supplementary file 1 [file diagnostics-11-01383-s001.zip › diagnostics-1261965-supplementary.pdf]

A. Fever

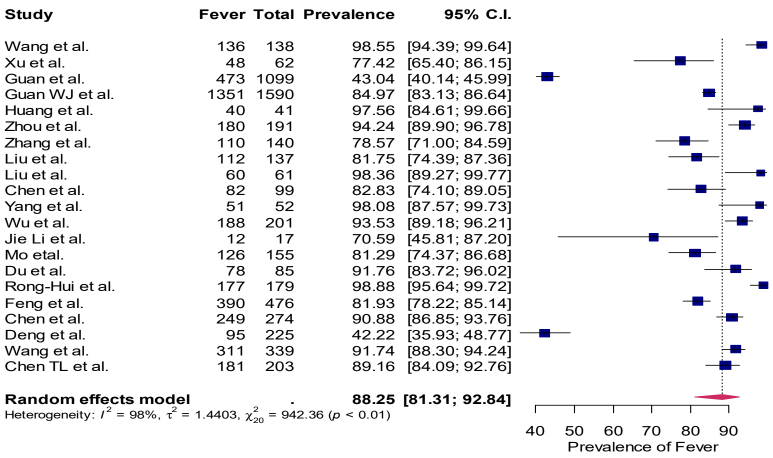

B. Cough

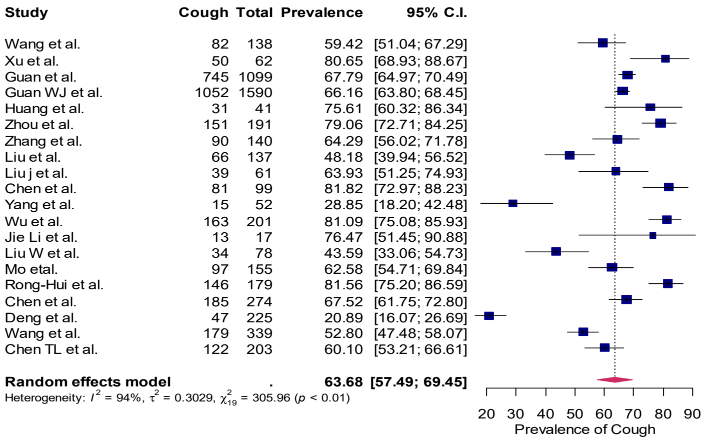

C. Fatigue

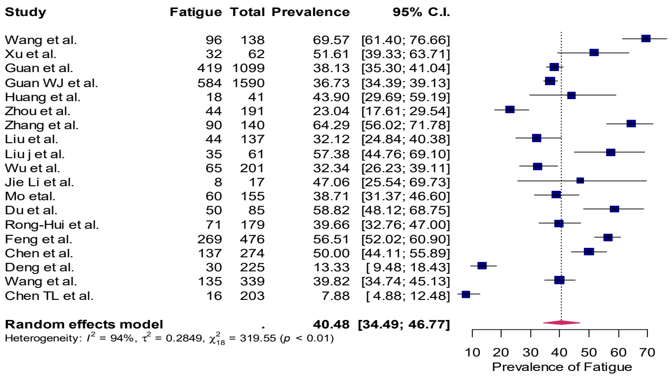

D. Anorexia

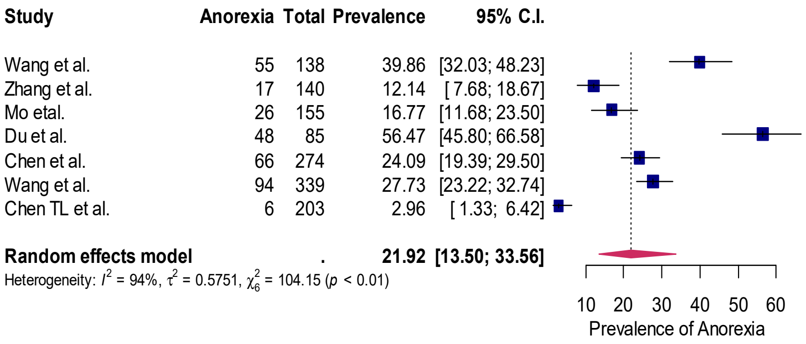

E. Myalgia

F. Dyspnea

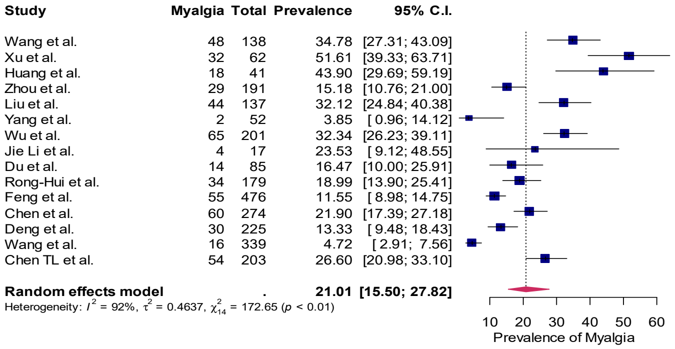

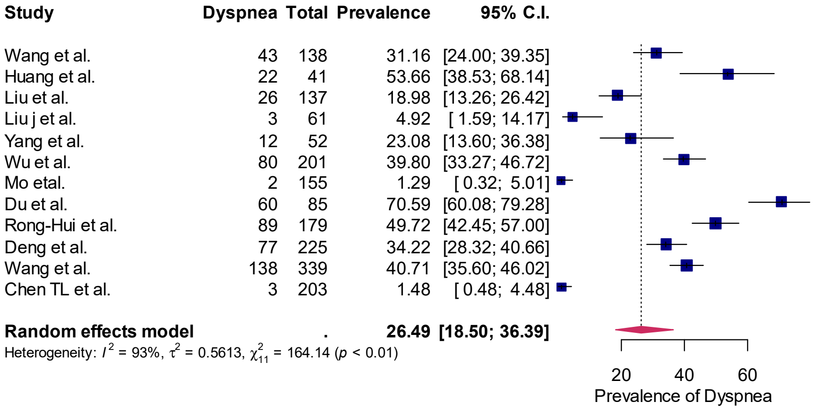

G. Diarrhea

H. Nausea

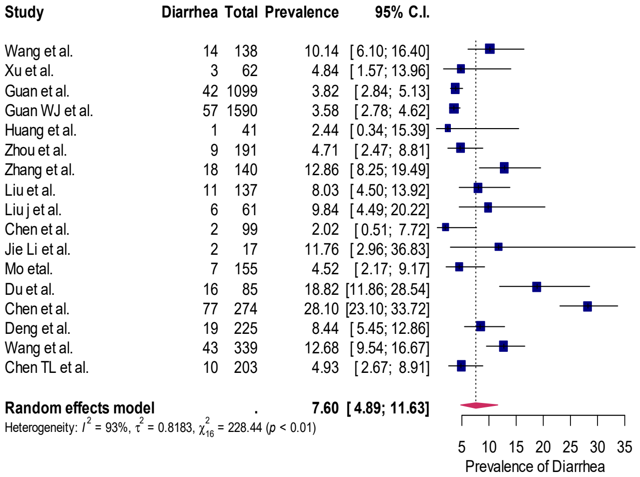

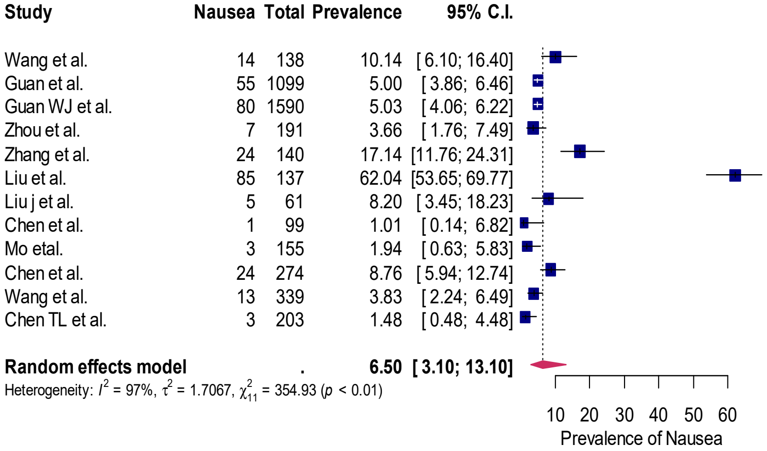

I. Headache

J. Hypertension

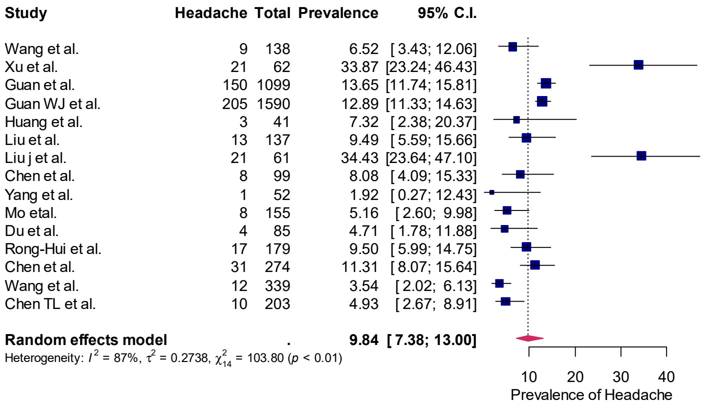

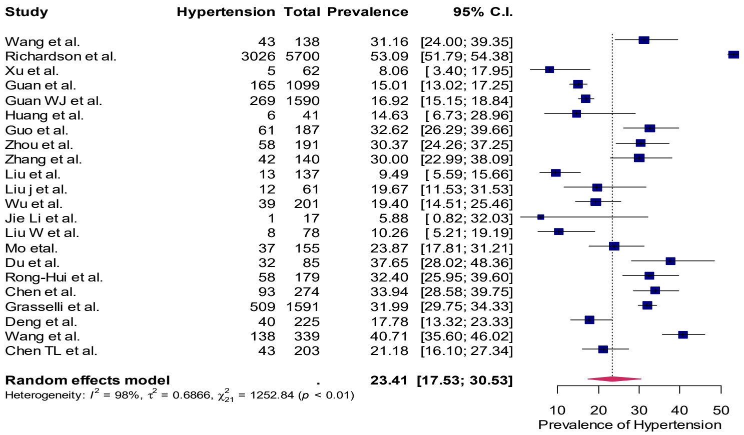

K. Diabetes

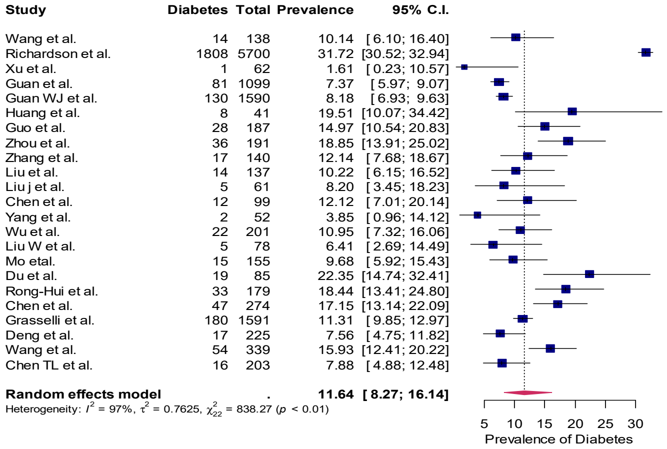

L. CVD

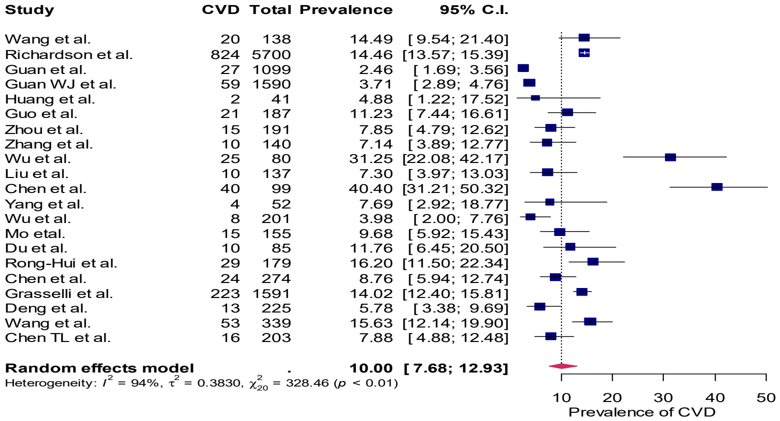

M. Malignancy

N. COPD

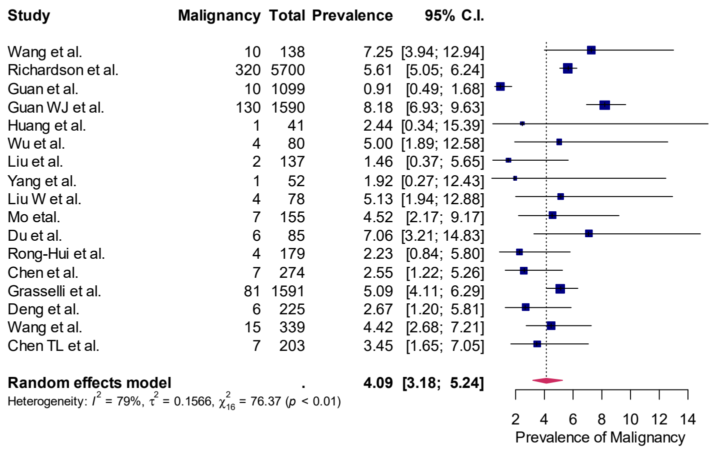

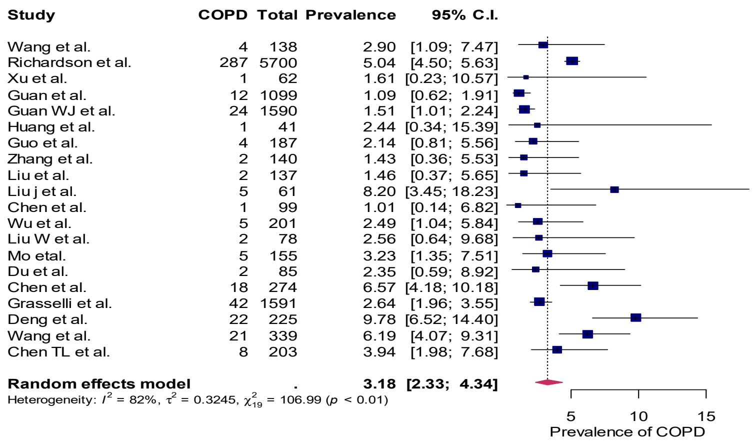

O. CERD

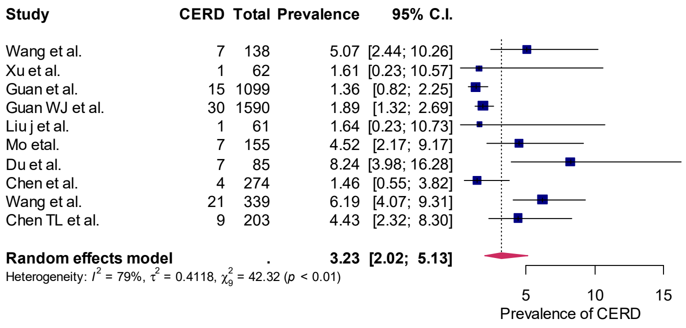

P. CKD

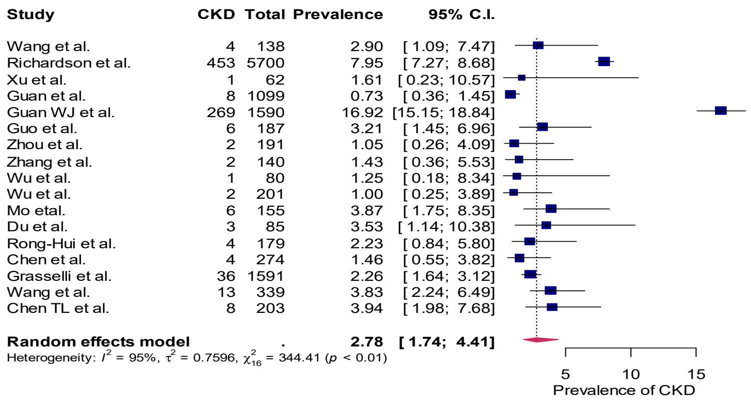

Q. CLD

R. Smoking

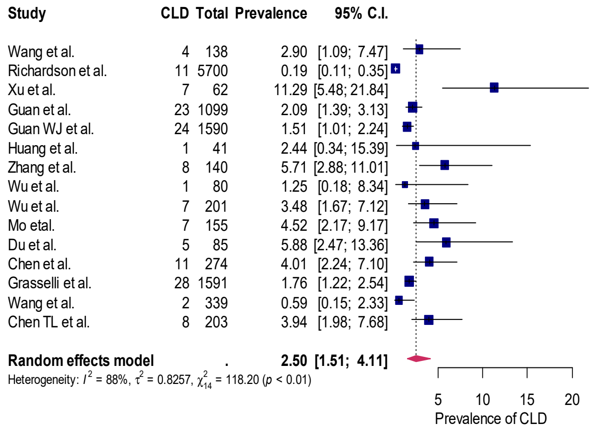

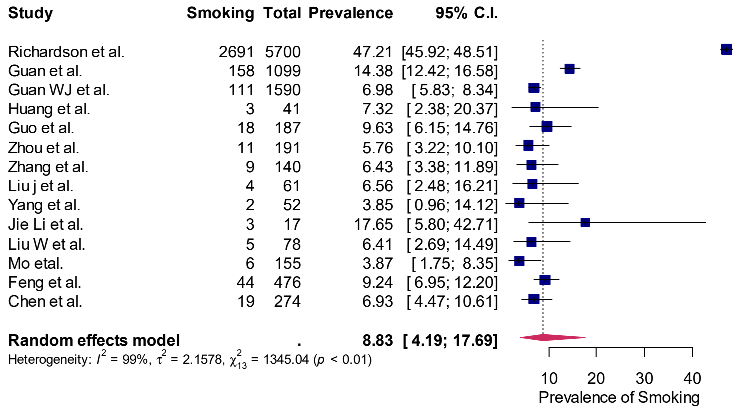

Supplementary Figure S1: Meta-analysis of prevalence of comorbidities and symptoms COVID-19 fatalities.

A. Fever

B. Cough

Study	Experimental Events	Experimental Total	Control Events	Control Total	Weight	Odds Ratio MH, Random, 95% CI
Wang et al.	36	136	36	138	9.1%	1.02 [0.60; 1.75]
Guan et al.	82	473	173	1099	16.3%	1.12 [0.84; 1.50]
Huang et al.	13	40	13	41	4.0%	1.04 [0.41; 2.64]
Zhou et al.	51	180	66	191	11.4%	0.75 [0.48; 1.16]
Zhang et al.	51	110	58	140	9.8%	1.22 [0.74; 2.02]
Liu et al.	17	60	17	61	5.2%	1.02 [0.46; 2.26]
Yang et al.	31	51	52	52	0.5%	0.01 [0.00; 0.25]
Mo etal.	63	126	55	155	10.4%	1.82 [1.13; 2.94]
Chen et al.	104	249	113	274	14.2%	1.02 [0.72; 1.45]
Wang et al.	56	311	65	339	12.7%	0.93 [0.62; 1.38]
Chen TL et al.	17	181	19	203	6.5%	1.00 [0.50; 2.00]
**Total (95% CI)**		**1917**		**2693**	**100.0%**	**1.04 [0.85; 1.28]**
**Prediction interval**						**[0.61; 1.79]**

Heterogeneity: $Tau^2 = 0.0459$; $Chi^2 = 17.34$, df = 10 (P = 0.07); $I^2 = 42\%$

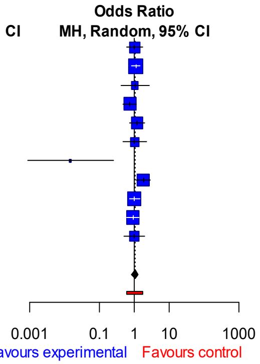

C. Fatigue

Study	Experimental Events	Experimental Total	Control Events	Control Total	Weight	Odds Ratio MH, Random, 95% CI
Wang et al.	21	82	36	138	8.0%	0.98 [0.52; 1.82]
Guan et al.	122	745	173	1099	19.4%	1.05 [0.81; 1.35]
Huang et al.	11	31	13	41	3.9%	1.18 [0.44; 3.18]
Zhou et al.	39	151	66	191	11.5%	0.66 [0.41; 1.06]
Zhang et al.	45	90	58	140	9.9%	1.41 [0.83; 2.41]
Liu et al.	14	39	17	61	4.9%	1.45 [0.61; 3.43]
Yang et al.	25	15	52	52	0.0%	
Mo etal.	54	97	55	155	10.2%	2.28 [1.36; 3.83]
Chen et al.	79	185	113	274	14.4%	1.06 [0.73; 1.55]
Wang et al.	30	179	65	339	11.3%	0.85 [0.53; 1.37]
Chen TL et al.	15	122	19	203	6.5%	1.36 [0.66; 2.78]
**Total (95% CI)**		**1736**		**2693**	**100.0%**	**1.12 [0.91; 1.38]**
**Prediction interval**						**[0.65; 1.92]**

Heterogeneity: $Tau^2 = 0.0430$; $Chi^2 = 15.20$, df = 9 (P = 0.09); $I^2 = 41\%$

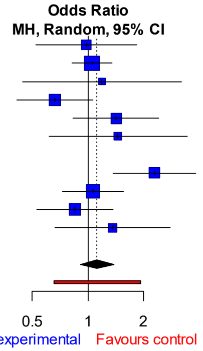

D. Anorexia

Study	Experimental Events	Experimental Total	Control Events	Control Total	Weight	Odds Ratio MH, Random, 95% CI
Wang et al.	29	96	36	138	8.7%	1.23 [0.69; 2.19]
Guan et al.	69	419	173	1099	31.4%	1.06 [0.78; 1.43]
Huang et al.	7	18	13	41	2.2%	1.37 [0.43; 4.34]
Zhou et al.	15	44	66	191	6.1%	0.98 [0.49; 1.96]
Zhang et al.	39	90	58	140	10.2%	1.08 [0.63; 1.85]
Liu j et al.	12	35	17	61	3.6%	1.35 [0.55; 3.30]
Mo etal.	27	60	55	155	8.0%	1.49 [0.81; 2.73]
Chen et al.	64	137	113	274	17.1%	1.25 [0.83; 1.89]
Wang et al.	26	135	65	339	11.4%	1.01 [0.61; 1.67]
Chen TL et al.	2	16	19	203	1.2%	1.38 [0.29; 6.55]
**Total (95% CI)**		**1050**		**2641**	**100.0%**	**1.14 [0.96; 1.36]**
**Prediction interval**						**[0.93; 1.40]**

Heterogeneity: $Tau^2 = 0$; $Chi^2 = 1.99$, df = 9 (P = 0.99); $I^2 = 0\%$

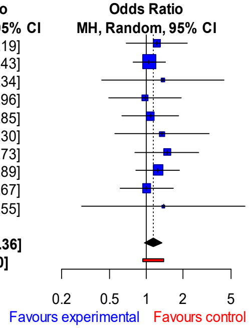

E. Myalgia

Study	Experimental Events	Experimental Total	Control Events	Control Total	Weight	Odds Ratio MH, Random, 95% CI
Wang et al.	24	55	36	138	21.7%	2.19 [1.14; 4.22]
Zhang et al.	8	17	58	140	14.7%	1.26 [0.46; 3.45]
Mo etal.	18	26	55	155	16.7%	4.09 [1.67; 10.02]
Chen et al.	31	66	113	274	24.4%	1.26 [0.74; 2.17]
Wang et al.	15	94	65	339	22.6%	0.80 [0.43; 1.48]
**Total (95% CI)**		**258**		**1046**	**100.0%**	**1.56 [0.93; 2.62]**
**Prediction interval**						**[0.29; 8.45]**

Heterogeneity: $Tau^2 = 0.2118$; $Chi^2 = 10.61$, df = 4 (P = 0.03); $I^2 = 62\%$

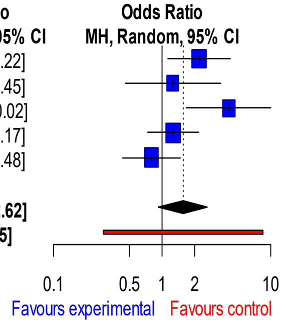

F. Dyspnea

Study	Experimental Events	Experimental Total	Control Events	Control Total	Weight	Odds Ratio MH, Random, 95% CI
Wang et al.	12	48	36	138	23.4%	0.94 [0.44; 2.01]
Huang et al.	7	18	13	41	10.1%	1.37 [0.43; 4.34]
Zhou et al.	8	29	66	191	17.8%	0.72 [0.30; 1.72]
Yang et al.	4	2	52	52	0.0%	
Chen et al.	21	60	113	274	39.5%	0.77 [0.43; 1.37]
Wang et al.	1	16	65	339	3.2%	0.28 [0.04; 2.17]
Chen TL et al.	2	54	19	203	6.0%	0.37 [0.08; 1.65]
**Total (95% CI)**		**227**		**1238**	**100.0%**	**0.78 [0.54; 1.13]**
**Prediction interval**						**[0.47; 1.31]**

Heterogeneity: $Tau^2 = 0$; $Chi^2 = 3.14$, df = 5 (P = 0.68); $I^2 = 0\%$

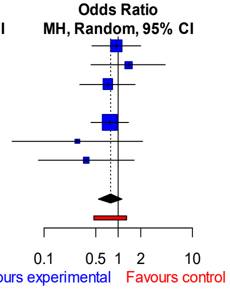

G. Diarrhea

Study	Experimental Events	Experimental Total	Control Events	Control Total	Weight	Odds Ratio MH, Random, 95% CI
Wang et al.	23	43	36	138	29.0%	3.26 [1.60; 6.62]
Huang et al.	12	22	13	41	15.8%	2.58 [0.89; 7.51]
Liu j et al.	3	3	17	61	2.4%	17.80 [0.87; 362.69]
Yang et al.	21	12	52	52	0.0%	
Mo etal.	2	2	55	155	2.3%	9.05 [0.43; 191.95]
Wang et al.	38	138	65	339	47.0%	1.60 [1.01; 2.54]
Chen TL et al.	1	3	19	203	3.5%	4.84 [0.42; 55.91]
**Total (95% CI)**		**223**		**989**	**100.0%**	**2.43 [1.52; 3.89]**
**Prediction interval**						**[0.92; 6.45]**

Heterogeneity: $Tau^2 = 0.0664$; $Chi^2 = 6.17$, df = 5 (P = 0.29); $I^2 = 19\%$

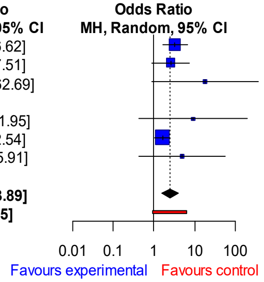

H. Nausea

Study	Experimental Events	Experimental Total	Control Events	Control Total	Weight	Odds Ratio MH, Random, 95% CI
Wang et al.	6	14	36	138	8.7%	2.12 [0.69; 6.54]
Guan et al.	10	42	173	1099	19.0%	1.67 [0.81; 3.47]
Zhou et al.	2	9	66	191	4.4%	0.54 [0.11; 2.68]
Zhang et al.	9	18	58	140	11.1%	1.41 [0.53; 3.78]
Liu j et al.	1	6	17	61	2.3%	0.52 [0.06; 4.76]
Mo etal.	5	7	55	155	4.1%	4.55 [0.85; 24.21]
Chen et al.	27	77	113	274	32.2%	0.77 [0.45; 1.30]
Wang et al.	8	43	65	339	15.6%	0.96 [0.43; 2.18]
Chen TL et al.	1	10	19	203	2.6%	1.08 [0.13; 8.96]
**Total (95% CI)**		**226**		**2600**	**100.0%**	**1.14 [0.81; 1.61]**
**Prediction interval**						**[0.66; 1.96]**

Heterogeneity: $Tau^2 = 0.0224$; $Chi^2 = 8.68$, df = 8 (P = 0.37); $I^2 = 8\%$

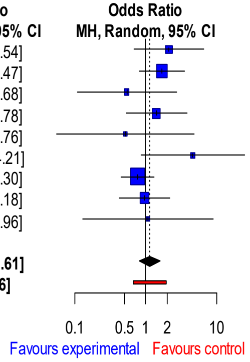

I. Headache

Study	Experimental Events	Experimental Total	Control Events	Control Total	Weight	Odds Ratio MH, Random, 95% CI
Wang et al.	4	14	36	138	12.3%	1.13 [0.33; 3.84]
Guan et al.	12	55	173	1099	31.9%	1.49 [0.77; 2.89]
Zhou et al.	3	7	66	191	8.3%	1.42 [0.31; 6.54]
Zhang et al.	5	24	58	140	16.1%	0.37 [0.13; 1.05]
Liu j et al.	2	5	17	61	5.6%	1.73 [0.26; 11.25]
Chen et al.	8	24	113	274	21.0%	0.71 [0.29; 1.72]
Wang et al.	1	13	65	339	4.7%	0.35 [0.04; 2.75]
**Total (95% CI)**		**142**		**2242**	**100.0%**	**0.93 [0.58; 1.47]**
**Prediction interval**						**[0.39; 2.20]**

Heterogeneity: $Tau^2 = 0.0589$; $Chi^2 = 7.07$, df = 6 (P = 0.31); $I^2 = 15\%$

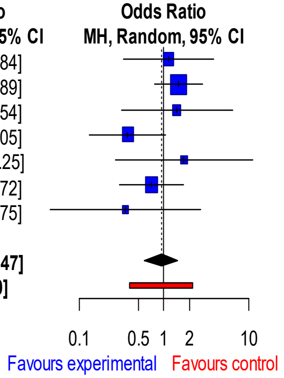

J. Hypertension

Study	Experimental Events	Experimental Total	Control Events	Control Total	Weight	Odds Ratio MH, Random, 95% CI
Wang et al.	3	9	36	138	7.6%	1.42 [0.34; 5.96]
Guan et al.	26	150	173	1099	53.1%	1.12 [0.71; 1.77]
Liu j et al.	3	21	17	61	8.6%	0.43 [0.11; 1.65]
Yang et al.	2	1	52	52	0.0%	
Mo etal.	5	8	55	155	7.3%	3.03 [0.70; 13.16]
Chen et al.	11	31	113	274	23.4%	0.78 [0.36; 1.70]
**Total (95% CI)**		**220**		**1779**	**100.0%**	**1.04 [0.69; 1.56]**
**Prediction interval**						**[0.45; 2.41]**

Heterogeneity: $Tau^2 = 0.0272$; $Chi^2 = 4.49$, df = 4 (P = 0.34); $I^2 = 11\%$

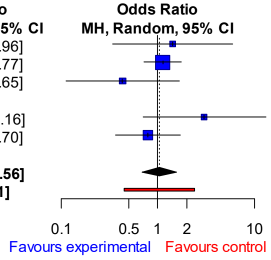

K. Diabetes

Study	Experimental Events	Experimental Total	Control Events	Control Total	Weight	Odds Ratio MH, Random, 95% CI
Wang et al.	21	43	36	138	7.6%	2.70 [1.33; 5.49]
Guan et al.	41	165	173	1099	25.1%	1.77 [1.20; 2.61]
Huang et al.	2	6	13	41	1.1%	1.08 [0.17; 6.65]
Zhou et al.	26	58	66	191	10.6%	1.54 [0.85; 2.80]
Zhang et al.	22	42	58	140	7.9%	1.56 [0.78; 3.11]
Liu j et al.	6	12	17	61	2.4%	2.59 [0.73; 9.15]
Mo etal.	22	37	55	155	7.0%	2.67 [1.28; 5.56]
Chen et al.	54	93	113	274	16.7%	1.97 [1.22; 3.18]
Wang et al.	32	138	65	339	16.5%	1.27 [0.79; 2.05]
Chen TL et al.	9	43	19	203	5.0%	2.56 [1.07; 6.14]
**Total (95% CI)**		**637**		**2641**	**100.0%**	**1.81 [1.49; 2.20]**
**Prediction interval**						**[1.44; 2.27]**

Heterogeneity: $Tau^2 = 0$; $Chi^2 = 6.22$, df = 9 (P = 0.72); $I^2 = 0\%$

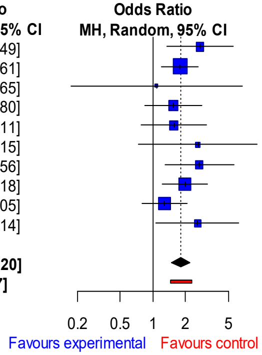

L. CVD

Study	Experimental Events	Experimental Total	Control Events	Control Total	Weight	Odds Ratio MH, Random, 95% CI
Wang et al.	8	14	36	138	8.3%	3.78 [1.23; 11.63]
Guan et al.	28	81	173	1099	17.9%	2.83 [1.74; 4.60]
Huang et al.	1	8	13	41	2.9%	0.31 [0.03; 2.77]
Zhou et al.	17	36	66	191	13.6%	1.69 [0.83; 3.48]
Zhang et al.	8	17	58	140	9.5%	1.26 [0.46; 3.45]
Liu j et al.	3	5	17	61	3.8%	3.88 [0.60; 25.31]
Yang et al.	7	2	52	52	0.0%	
Mo etal.	12	15	55	155	6.8%	7.27 [1.97; 26.88]
Chen et al.	24	47	113	274	15.4%	1.49 [0.80; 2.76]
Wang et al.	11	54	65	339	13.7%	1.08 [0.53; 2.21]
Chen TL et al.	5	16	19	203	8.0%	4.40 [1.38; 14.01]
**Total (95% CI)**		**295**		**2693**	**100.0%**	**2.08 [1.39; 3.10]**
**Prediction interval**						**[0.72; 6.01]**

Heterogeneity: $Tau^2 = 0.1703$; $Chi^2 = 16.77$, df = 9 (P = 0.05); $I^2 = 46\%$

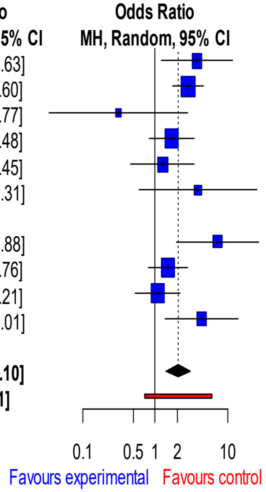

M. Malignancy

Study	Experimental Events	Experimental Total	Control Events	Control Total	Weight	Odds Ratio MH, Random, 95% CI
Wang et al.	9	20	36	138	20.5%	2.32 [0.89; 6.05]
Huang et al.	3	2	13	41	0.0%	
Mo etal.	14	15	55	155	6.0%	25.45 [3.26; 198.77]
Chen et al.	16	24	113	274	22.9%	2.85 [1.18; 6.88]
Wang et al.	21	53	65	339	34.2%	2.77 [1.50; 5.11]
Chen TL et al.	6	16	19	203	16.5%	5.81 [1.90; 17.75]
**Total (95% CI)**		**130**		**1150**	**100.0%**	**3.46 [2.05; 5.87]**
**Prediction interval**						**[0.88; 13.63]**

Heterogeneity: $Tau^2 = 0.1131$; $Chi^2 = 5.88$, df = 4 (P = 0.21); $I^2 = 32\%$

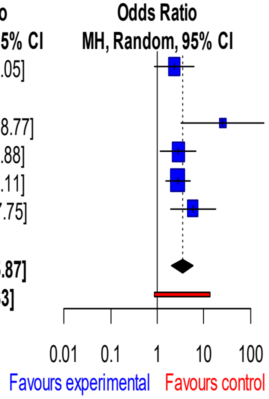

N. COPD

Study	Experimental Events	Experimental Total	Control Events	Control Total	Weight	Odds Ratio MH, Random, 95% CI
Wang et al.	4	10	36	138	27.2%	1.89 [0.50; 7.08]
Yang et al.	1	1	52	52	0.0%	
Mo etal.	5	7	55	155	17.0%	4.55 [0.85; 24.21]
Chen et al.	5	7	113	274	17.3%	3.56 [0.68; 18.68]
Wang et al.	3	15	65	339	28.4%	1.05 [0.29; 3.84]
Chen TL et al.	1	7	19	203	10.1%	1.61 [0.18; 14.12]
**Total (95% CI)**		**47**		**1161**	**100.0%**	**2.04 [1.02; 4.07]**
**Prediction interval**						**[0.67; 6.25]**

Heterogeneity: $Tau^2 = 0$; $Chi^2 = 2.38$, df = 4 (P = 0.67); $I^2 = 0\%$

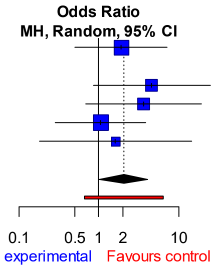

O. CERD

Study	Experimental Events	Experimental Total	Control Events	Control Total	Weight	Odds Ratio MH, Random, 95% CI
Wang et al.	3	4	36	138	6.1%	8.50 [0.86; 84.34]
Guan et al.	6	12	173	1099	24.7%	5.35 [1.71; 16.79]
Huang et al.	1	1	13	41	3.0%	6.33 [0.24; 165.88]
Zhang et al.	2	2	58	140	3.5%	7.05 [0.33; 149.60]
Liu j et al.	3	5	17	61	9.2%	3.88 [0.60; 25.31]
Mo etal.	4	5	55	155	6.6%	7.27 [0.79; 66.69]
Wang et al.	11	21	65	339	40.0%	4.64 [1.89; 11.38]
Chen TL et al.	1	8	19	203	7.0%	1.38 [0.16; 11.85]
**Total (95% CI)**		**58**		**2176**	**100.0%**	**4.76 [2.69; 8.39]**
**Prediction interval**						**[2.34; 9.66]**

Heterogeneity: $Tau^2 = 0$; $Chi^2 = 1.84$, df = 7 (P = 0.97); $I^2 = 0\%$

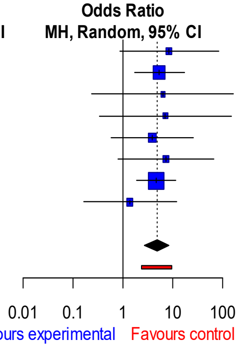

P. CKD

Study	Experimental Events	Experimental Total	Control Events	Control Total	Weight	Odds Ratio MH, Random, 95% CI
Wang et al.	6	7	36	138	9.2%	17.00 [1.98; 146.06]
Guan et al.	4	15	173	1099	25.9%	1.95 [0.61; 6.18]
Mo etal.	7	7	55	155	5.3%	27.16 [1.52; 484.56]
Chen et al.	4	4	113	274	5.1%	12.81 [0.68; 240.21]
Wang et al.	10	21	65	339	36.6%	3.83 [1.56; 9.41]
Chen TL et al.	3	9	19	203	17.9%	4.84 [1.12; 20.94]
**Total (95% CI)**		**63**		**2208**	**100.0%**	**4.54 [2.29; 8.99]**
**Prediction interval**						**[1.16; 17.83]**

Heterogeneity: $Tau^2 = 0.1215$; $Chi^2 = 5.97$, df = 5 (P = 0.31); $I^2 = 16\%$

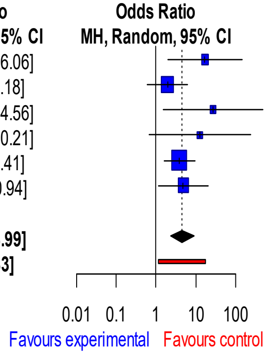

Study	Experimental Events	Experimental Total	Control Events	Control Total	Weight	Odds Ratio MH, Random, 95% CI
Wang et al.	2	4	36	138	10.3%	2.83 [0.38; 20.86]
Guan et al.	3	8	173	1099	19.7%	3.21 [0.76; 13.56]
Zhou et al.	2	2	66	191	4.4%	9.44 [0.45; 199.42]
Zhang et al.	2	2	58	140	4.4%	7.05 [0.33; 149.60]
Mo etal.	4	6	55	155	13.7%	3.64 [0.65; 20.49]
Chen et al.	4	4	113	274	4.8%	12.81 [0.68; 240.21]
Wang et al.	4	13	65	339	28.0%	1.87 [0.56; 6.27]
Chen TL et al.	2	8	19	203	14.7%	3.23 [0.61; 17.12]
**Total (95% CI)**		**47**		**2539**	**100.0%**	**3.22 [1.70; 6.10]**
**Prediction interval**						**[1.45; 7.15]**

Heterogeneity: $Tau^2 = 0$; $Chi^2 = 2.49$, df = 7 (P = 0.93); $I^2 = 0\%$

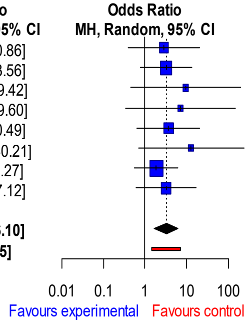

Q. Smoking

Study	Experimental Events	Experimental Total	Control Events	Control Total	Weight	Odds Ratio MH, Random, 95% CI
Guan et al.	38	158	173	1099	68.5%	1.69 [1.14; 2.53]
Zhou et al.	5	11	66	191	7.3%	1.58 [0.46; 5.37]
Zhang et al.	6	9	58	140	5.4%	2.83 [0.68; 11.77]
Liu j et al.	2	4	17	61	2.6%	2.59 [0.34; 19.87]
Mo etal.	4	6	55	155	3.7%	3.64 [0.65; 20.49]
Chen et al.	9	19	113	274	12.6%	1.28 [0.50; 3.26]
**Total (95% CI)**		**207**		**1920**	**100.0%**	**1.74 [1.25; 2.42]**
**Prediction interval**						**[1.09; 2.78]**

Heterogeneity: $Tau^2 = 0$; $Chi^2 = 1.74$, df = 5 (P = 0.88); $I^2 = 0\%$

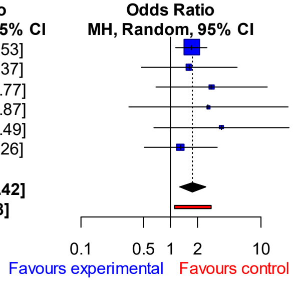

Supplementary Figure S2: Meta-analysis of severity of comorbidities and symptoms in COVID-19 fatalities

A. Fever **B. Cough** **C. Fatigue** **D. Dyspnea**

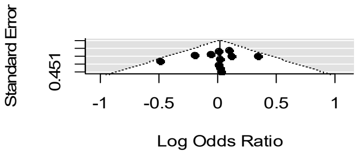

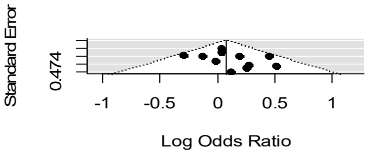

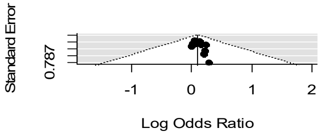

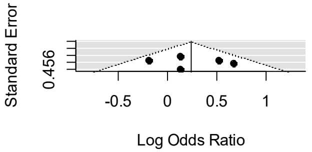

Egger's test: p= 0.479 **Egger's test: p= 0.354** **Egger's test: p=0.183** **Egger's test: p= 0.774**

E. Myalgia **F. Anorexia** **G. Diarrhea** **H. Nausea**

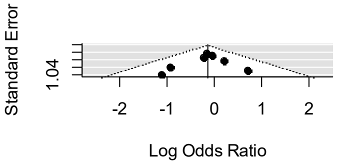

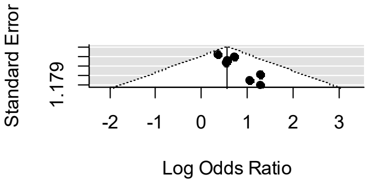

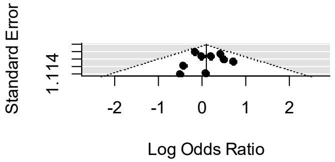

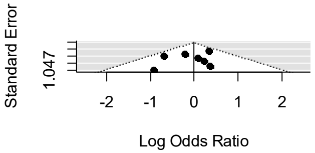

Egger's test: p= 0.685 **Egger's test: p=0.018** **Egger's test: p= 0.731** **Egger's test: p= 0.458**

I. Headache **J. Hypertension** **K. Diabetes** **L. CVD**

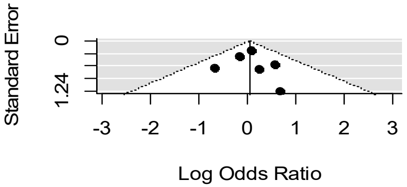

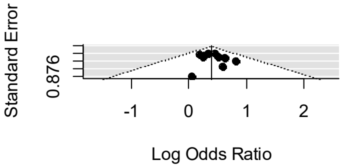

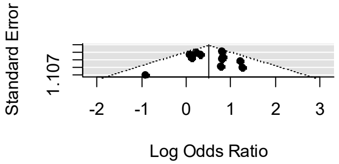

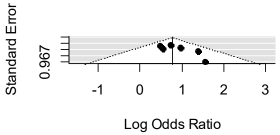

Egger's test: p= 0.832 **Egger's test: p=0.551** **Egger's test: p= 0.949** **Egger's test: p= 0.141**

M. Malignancy **N. CRED** **O. Smoking**

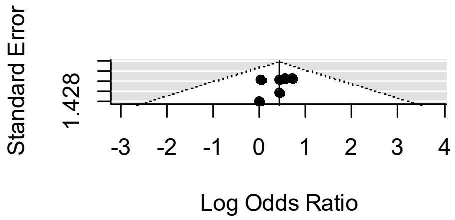

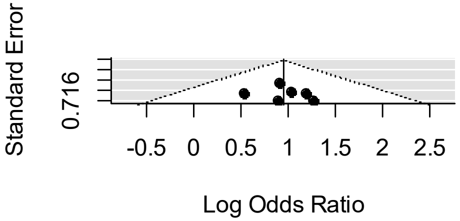

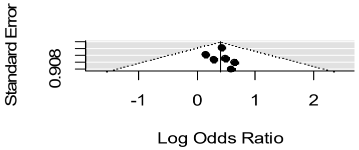

Egger's test: p=0.466

Egger's test: p= 0.633

Egger's test: p=0.916

P. CKD

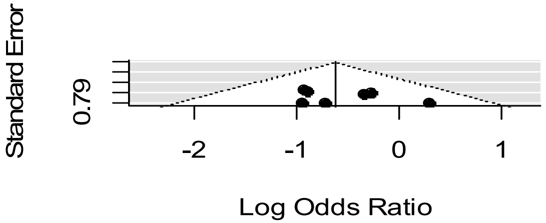

Egger's test: p= 0.593

Q. COPD

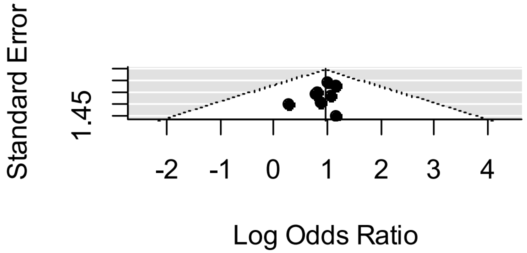

Egger's test: p=0.235

Supplementary Figure S3: Assessment of publication bias using funnel plot and Egger's test

Supplementary Table S1: Accuracy and Evaluation matrices for symptoms data in ML analysis

Algorithms	Accuracy	Precision	Recall	f1 Score	AUC	Log loss
Random Forest	0.87	0.89	0.93	0.91	0.83	4.52
Decision Tree	0.87	0.9	0.93	0.91	0.84	4.37
XGB	0.89	0.92	0.93	0.92	0.87	3.77
GBM	0.9	0.93	0.94	0.93	0.88	3.32
SVM	0.89	0.93	0.91	0.92	0.87	3.92
Light GBM	0.9	0.94	0.91	0.93	0.89	3.47

Note: XGB= XGBoost; GBM= Gradient Boosting Machine; SVM= Support Vector Machine

Supplementary Table S2: Accuracy and Evaluation matrices for comorbidity data in ML analysis

Algorithms	Accuracy	Precision	Recall	F1 Score	AUC	Log loss
**Random Forest**	0.87	0.89	0.93	0.91	0.82	4.52
**Decision Tree**	0.83	0.85	0.92	0.88	0.76	5.88
**XGB**	0.86	0.89	0.92	0.9	0.82	4.83
**GBM**	0.84	0.92	0.86	0.89	0.83	5.43
**SVM**	0.87	0.91	0.91	0.91	0.84	4.52
**Light GBM**	0.88	0.9	0.93	0.92	0.84	4.22

Note: XGB= XGBoost; GBM= Gradient Boosting Machine; SVM= Support Vector Machine

Supplementary Table S3: Coefficient values for each symptom applying after ML methods

Algorithms	Headache	Fever	Cough	Fatigue	Nausea	Diarrhea	Myalgia	Dyspnea	Pneumonia	ARDS	Septic Shock
Random Forest	0.3	1.89	1.74	0.61	0.18	0.16	0.71	2.94	76.62	4.77	0.69
Decision Tree	0.28	2.19	1.28	0.9	0.4	0.07	0.68	1.78	4.3	3.66	0.17
XGB	0	6.67	6.74	3.81	0	0	3.2	13.34	19.7	25.4	1.92
GBM	0.74	1.6	0.95	0.34	0.07	0.04	0.56	3.07	9.18	7.22	0.8
SVM	0	24.53	25.3	10.7	10.7	10.7	8.22	21.74	58.67	42.5	5.52
Light GBM	0	39.77	21.02	0	0	0	0	34.84	95.68	37.7	0.16

Note: ARDS= Acute respiratory distress syndrome

Supplementary Table S4: Coefficient values for each comorbidity applying after ML methods

Algorithms	Gender	Age	Hypertension	CVD	CEVD	Chronic Lung Disease	Malignancy	Diabetes and Metabolic Disease	CLD	CKD	Neurodegenerative Disease	Infectious Disease	Surgical History	COPD	Asthma
**Random Forest**	2.56	82.9	5.53	1.06	0.27	0.35	0.65	3.67	0.13	1.13	0.27	0.03	0.43	0.82	0.19
**Decision Tree**	4.05	85.51	2.57	0.49	0.28	0.45	0.88	3.77	0	0.81	0	0.007	0.55	0.52	0.1
**XGB**	9.37	20.77	14.84	5.98	0	2.9	7.37	14.21	0	7	0	0	0	5.77	11.8
**GBM**	2.19	77.78	6.48	1.37	0.27	0.23	0.73	7.72	0.09	0.87	0.46	0.05	0.64	1.02	0.09
**SVM**	4.07	169.3	34	10.18	23.89	11.66	0	33.58	22.7	29.2	14	15.78	8.34	25.4	24.15
**Light GBM**	54.6	1448.16	114.62	0.81	0	0	0	46.38	0	3.53	0	0	0	0	0

Note: CVD= Cardiovascular disease; COPD=Chronic obstructive pulmonary disease; CEVD= Cerebrovascular disease; CKD=Chronic Kidney Disease; CLD= Chronic lung disease;

Supplementary Table S5: Assessing association between comorbidity and symptoms using Fisher's exact test of deceased patients

Comorbidity	Symptoms	P value	Comorbidity	Symptoms	P value
**Hypertension**	Headache	0.232	**CEVD**	Headache	1.00
	Fever	0.423		Fever	0.408
	Cough	0.823		Cough	0.318
	Fatigue	0.665		Fatigue	1.00
	Nausea or vomiting	0.232		Nausea or vomiting	1.00
	Diarrhea	1.00		Diarrhea	1.00
	Myalgia or arthralgia	1.00		Myalgia or arthralgia	0.037
	Dyspnea	<0.001		Dyspnea	1.00
	Pneumonia	<0.001		Pneumonia	0.191
	ARDS	<0.001		ARDS	1.00
	Septic Shock	<0.001		Septic Shock	0.187
**Diabetes**	Headache	0.179	**CLD**	Headache	1.00
	Fever	0.374		Fever	1.00
	Cough	0.444		Cough	0.008
	Fatigue	0.613		Fatigue	1.00
	Nausea or vomiting	1.00		Nausea or vomiting	1.00
	Diarrhea	0.179		Diarrhea	1.00
	Myalgia or arthralgia	0.447		Myalgia or arthralgia	1.00
	Dyspnea	<0.001		Dyspnea	0.022
	Pneumonia	<0.001		Pneumonia	1.00
	ARDS	<0.001		ARDS	1.00
	Septic Shock	0.047		Septic Shock	1.00
**CVD**	Headache	1.00	**Malignancy**	Headache	1.00
	Fever	0.117		Fever	1.00
	Cough	0.169		Cough	1.00
	Fatigue	1.00		Fatigue	0.085
	Nausea or vomiting	1.00		Nausea or vomiting	1.00
	Diarrhea	1.00		Diarrhea	1.00
	Myalgia or arthralgia	1.00		Myalgia or arthralgia	1.00
	Dyspnea	0.075		Dyspnea	1.00
	Pneumonia	0.001		Pneumonia	0.191
	ARDS	0.017		ARDS	0.567
	Septic Shock	0.039		Septic Shock	0.187

Supplementary Table S5: Assessing association between comorbidity and symptoms using Fisher's exact test of deceased patients (continued…)

Comorbidity	Symptoms	P value	Comorbidity	Symptoms	P value
**Liver Disease**	Headache	1.00	**Surgical History**	Headache	1.00
	Fever	1.00		Fever	1.00
	Cough	0.008		Cough	1.00
	Fatigue	1.00		Fatigue	0.022
	Nausea or vomiting	1.00		Nausea or vomiting	1.00
	Diarrhea	1.00		Diarrhea	1.00
	Myalgia or arthralgia	1.00		Myalgia or arthralgia	1.00
	Dyspnea	0.022		Dyspnea	1.00
	Pneumonia	1.00		Pneumonia	1.00
	ARDS	1.00		ARDS	1.00
	Septic Shock	1.00		Septic Shock	1.00
**COPD**	Headache	0.019	**Asthma**	Headache	1.00
	Fever	0.546		Fever	0.481
	Cough	0.438		Cough	0.381
	Fatigue	0.126		Fatigue	1.00
	Nausea or vomiting	1.00		Nausea or vomiting	1.00
	Diarrhea	1.00		Diarrhea	1.00
	Myalgia or arthralgia	1.00		Myalgia or arthralgia	1.00
	Dyspnea	0.224		Dyspnea	0.1645
	Pneumonia	0.018		Pneumonia	0.062
	ARDS	0.083		ARDS	<0.001
	Septic Shock	0.268		Septic Shock	0.2281
**Neurodegenerative Disease**	Headache	1.00	**CKD**	Headache	0.056
	Fever	0.015		Fever	0.708
	Cough	0.008		Cough	1.00
	Fatigue	0.043		Fatigue	0.337
	Nausea or vomiting	1.00		Nausea or vomiting	1.00
	Diarrhea	1.00		Diarrhea	1.00
	Myalgia or arthralgia	1.00		Myalgia or arthralgia	1.00
	Dyspnea	0.022		Dyspnea	0.164
	Pneumonia	1.00		Pneumonia	<0.001
	ARDS	1.00		ARDS	<0.001
	Septic Shock	1.00		Septic Shock	0.0087

Supplementary Table S6: The distribution of patients' according to countries

Country	Count
Italy	158090
USA	122453
China	42531
Russia	32761
UK	17837
Spain	16273
Ecuador	10947
France	10160
South Korea	7198
Iran	7044
Germany	6684
Egypt	4636

South Africa	4542
Austria	4242
Netherlands	4150
Brazil	4021
Belgium	3747
Chile	2739
Mexico	2112
Portugal	2063
Japan	2035
Canada	1721
Philippines	1512
Algeria	1268
Argentina	1137
Cuba	854
Niger	766
Switzerland	732
Nigeria	668
Norway	658
Sweden	637
Australia	561
Singapore	430
Uruguay	415
Colombia	377
Ghana	367
Kenya	337
Poland	325
Kazakhstan	302
Denmark	250
Thailand	119
Paraguay	113
Hong Kong	102
Ethiopia	98
Kuwait	93
Bahrain	85
Bolivia	81
Puerto Rico	69
Georgia	68
United Arab Emirates	61
Malaysia	57

Oman	55
Rwanda	54
Vietnam	50
Guatemala	50
Malta	49
India	40
Iraq	36
Taiwan	34
Iceland	34
Zimbabwe	30
Greece	29
Romania	25
Israel	23
Qatar	18
Reunion	18
Ireland	14
Namibia	14
Lebanon	13
Pakistan	12
Senegal	12
Peru	10
New Zealand	10
Afghanistan	9
Finland	8
Saudi Arabia	8
Croatia	7
Gambia	6
Azerbaijan	6
Guyana	5
Kosovo	5
Benin	5
Czech Republic	4
Morocco	4
Maldives	4
Cameroon	4
Burkina Faso	4
Nepal	3
Sri Lanka	3
Ukraine	3

Luxembourg	3
Cambodia	3
San Marino	2
Tunisia	2
Jordan	2
Bosnia	2
Congo	2
Venezuela	2
Sudan	2
Guinea	2
Armenia	2
Bhutan	2
Panama	2
Mongolia	2
Virgin Islands, U.S.	2
Moldova	1
North Macedonia	1
Estonia	1
Lithuania	1
Latvia	1
Andorra	1
Liechtenstein	1
Serbia	1
Gibraltar	1
Slovenia	1
Saint Vincent	1
